# Supplementary material for: A pleurocidin analogue with greater conformational flexibility, enhanced antimicrobial potency and in vivo therapeutic efficacy
Source: Commun Biol. 2020 Nov 27;3:697. doi: 10.1038/s42003-020-01420-3 (PMC7699649; doi:10.1038/s42003-020-01420-3)
Supplement: Supplementary file 1 — Supplementary Information [file 42003_2020_1420_MOESM1_ESM.pdf]

# A Pleurocidin Analogue with Greater Conformational Flexibility, Enhanced Antimicrobial Potency and *in vivo* Therapeutic Efficacy

Giorgia Manzo[1†], Charlotte, K. Hind[2†], Philip M. Ferguson[1†], Richard T. Amison[1,3†], Alice C. Hodgson-Casson[1], Katarzyna A. Ciazynska[1], Bethany J. Weller[1], Maria Clarke[1], Carolyn Lam[1], Rico C.H. Man[4], Blaze G. O'Shaughnessy[1,3], Melanie Clifford[2], Tam T. Bui[5], Alex F. Drake[5], R. Andrew Atkinson[5], Jenny K.W. Lam[4], Simon C. Pitchford[1,3], Clive P. Page[1,3], David A. Phoenix[6], Christian D. Lorenz[6]*, J. Mark Sutton[2]* and A. James Mason[1]*

[1]Institute of Pharmaceutical Science, School of Cancer & Pharmaceutical Science, King's College London, Franklin-Wilkins Building, 150 Stamford Street, London, SE1 9NH, United Kingdom

[2]Technology Development Group, National Infection Service, Public Health England, Salisbury, UK

[3]Sackler Institute of Pulmonary Pharmacology, King's College London

[4]Department of Pharmacology and Pharmacy, LKS Faculty of Medicine, The University of Hong Kong, Hong Kong

[5]Centre for Biomolecular Spectroscopy and Randall Division of Cell and Molecular Biophysics, King's College London, New Hunt's House, London SE1 1UL, United Kingdom

[6]School of Applied Science, London South Bank University, 103 Borough Road, London SE1 0AA, United Kingdom

[7]Department of Physics, King's College London, London WC2R 2LS, United Kingdom

# Supplementary Material

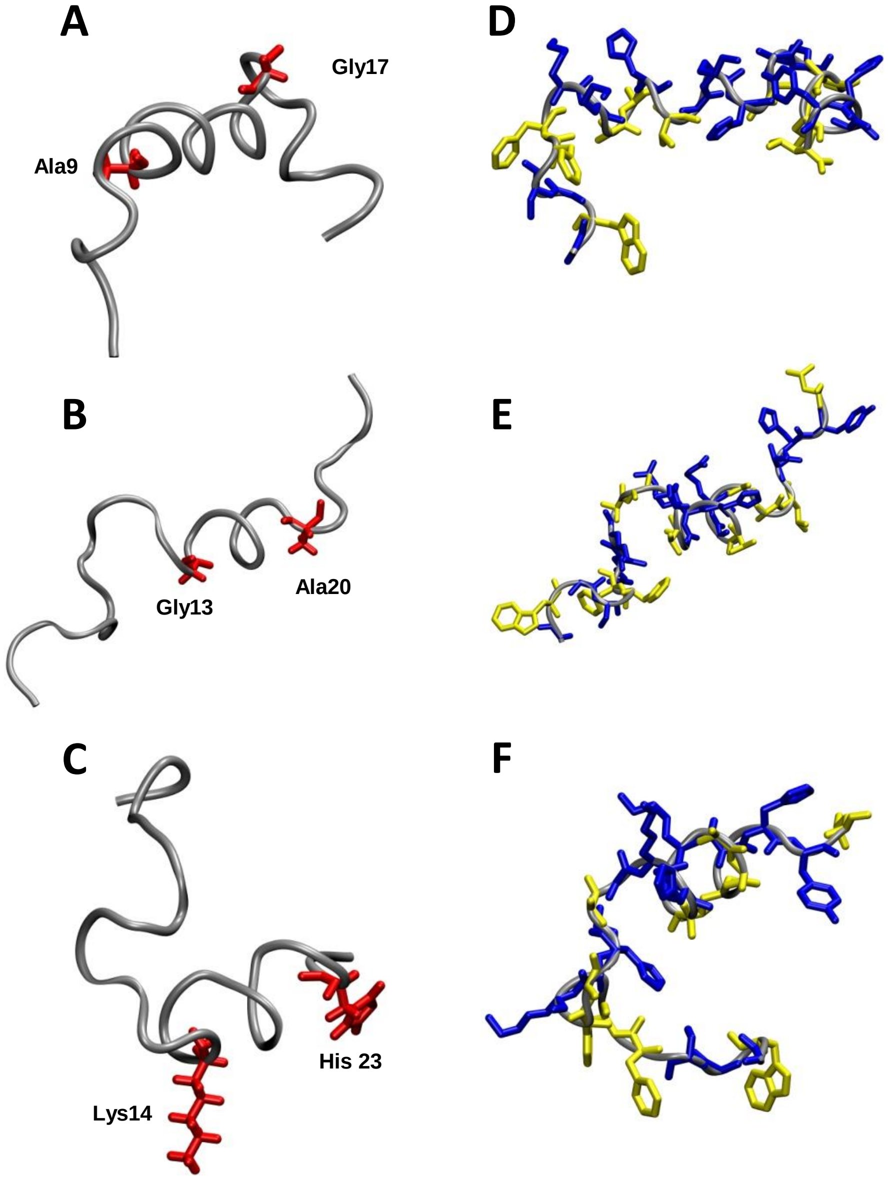


**Supplementary Figure 1. two representations of the three-dimensional structure for each of the three pleurocidin analogues.** Structures were determined through $^{1}$H-NOESY NMR spectroscopy in SDS-$d_{25}$ micelles (peptide/detergent equal to 1/50). The structure with the minimum RMSD is shown and was used as starting point in the MD simulations. First and last residues in helical conformation are highlighted in red for pleurocidin (A), pleurocidin-KR (B) and pleurocidin-VA (C). The hydrophobic and hydrophilic residues are shown in yellow and blue respectively for pleurocidin (D), pleurocidin-KR (E) and pleurocidin-VA (F).

**Supplementary Table 1 NMR and refinement statistics for pleurocidin-KR structure**
**Pairwise r.m.s. deviation was calculated among 50 refined structures.

| | Pleurocidin-KR | Pleurocidin-VA |
|---|---|---|
| **NMR distance and dihedral constraints** | | |
| Distance constraints | | |
| Total NOE | 83 | 100 |
| Intra-residue | 64 | 79 |
| Inter-residue | 22 | 21 |
| Sequential ($\|i - j\| = 1$) | 8 | 11 |
| Medium-range ($\|i - j\| < 4$) | 14 | 10 |
| Long-range ($\|i - j\| > 5$) | 0 | 0 |
| Intermolecular | 0 | 0 |
| Hydrogen bonds | 0 | 0 |
| Total dihedral angle restraints | 0 | 0 |
| $\phi$ | **0** | **0** |
| $\psi$ | **0** | **0** |
| | | |
| **Structure statistics** | | |
| Violations (mean and s.d.) | | |
| Distance constraints (Å) | 0.001 ± 0 | 0.0011 ± 0.0003 |
| Dihedral angle constraints (º) | 0 | 0 |
| Max. dihedral angle violation (º) | 0 | 0 |
| Max. distance constraint violation (Å) | 0.001 | 0.002 |
| Deviations from idealized geometry | | |
| Bond lengths (Å) | 0.00098 ± 0.00004 | $0.0011 \pm 4.90 \times 10^{-5}$ |
| Bond angles (º) | 0.1912 ± 0.0003 | $0.1999 \pm 4.50 \times 10^{-4}$ |
| Impropers (º) | 0.021 ± 0.003 | $0.0175 \pm 3.30 \times 10^{-3}$ |
| Average pairwise r.m.s. deviation** (Å) | | |
| Heavy | 4.89 ± 1.07 | 5.06 ± 0.87 |
| Backbone | 4.76 ± 1.05 | 5.17 ± 0.82 |

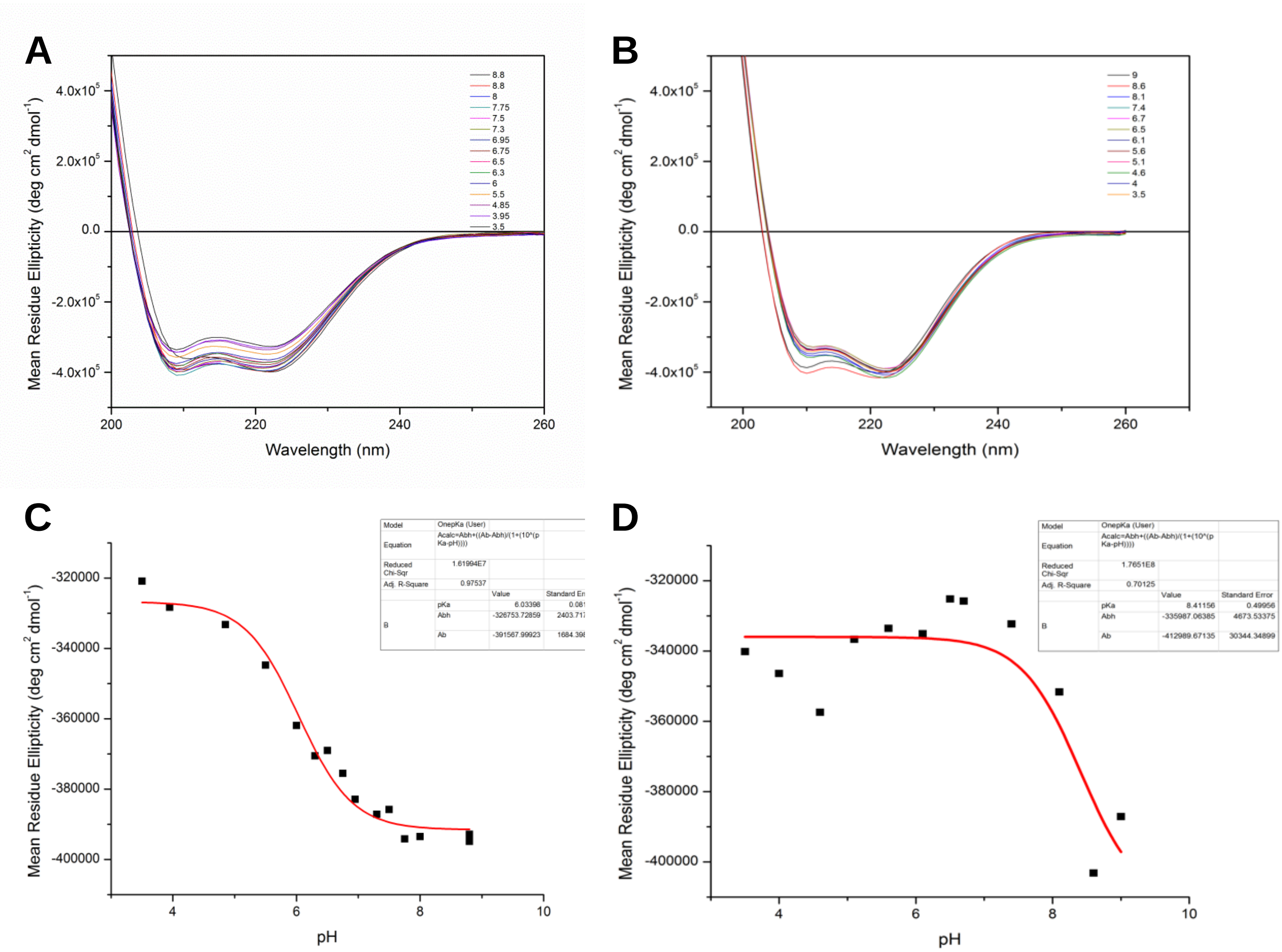


**Supplementary Figure 2. pH dependence of pleurocidin conformation.** The secondary structure of pleurocidin in POPE/POPG (A/C) or POPG (B/D) liposomes is shown with far-UV CD spectra (A/B) and the mean residue ellipticity at 220 nm (C/D) both shown as a function of pH. In POPE/POPG a pH dependent switch between more and less ordered α-helix conformations can be detected with a mid-point of the transition being pH 6.01 ± 0.02 (n = 2). No transition was detected in the sampled range for POPG only liposomes.

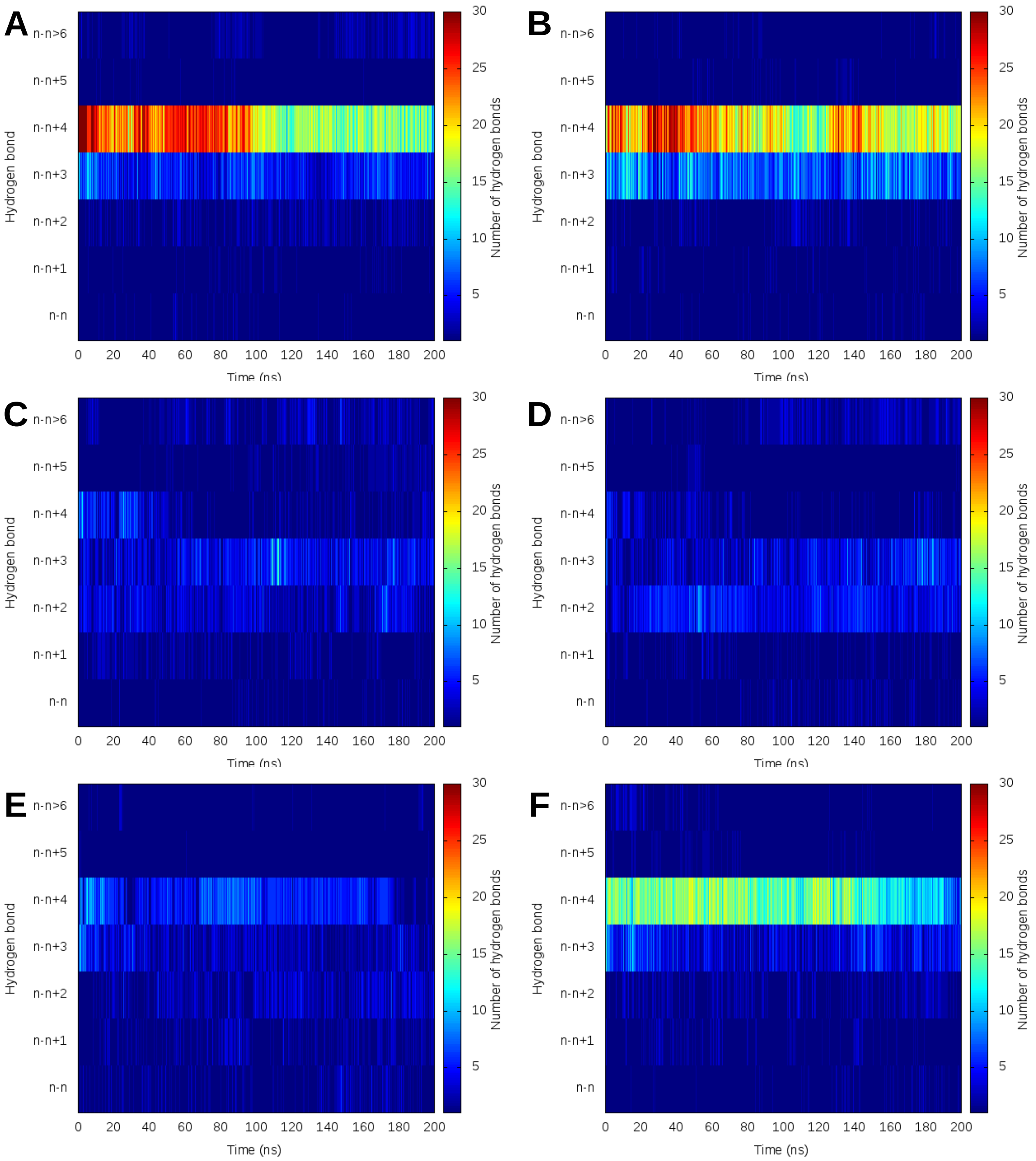


**Supplementary Figure 3. Secondary structure analysis of pleurocidin peptides from MD simulations (intramolecular hydrogen bonding).** Total intramolecular hydrogen bonds are shown as a function of time for pleurocidin and its two analogues in representative MD simulations (positively charged histidines); POPE/POPG bilayers (A/C/E), POPG bilayers (B/D/F) for pleurocidin (A/B), pleurocidin-KR (C/D) and pleurocidin-VA (E/F). Hydrogen bonding from residues n to n+4, consistent with α-helix or β-turn conformations, predominate in pleurocidin when binding to both bilayers. These are largely absent from pleurocidin-KR when binding to both bilayer types and pleurocidin-VA when binding to POPE/POPG but not POPG. Each panel represents an average of all four peptides in each simulation.

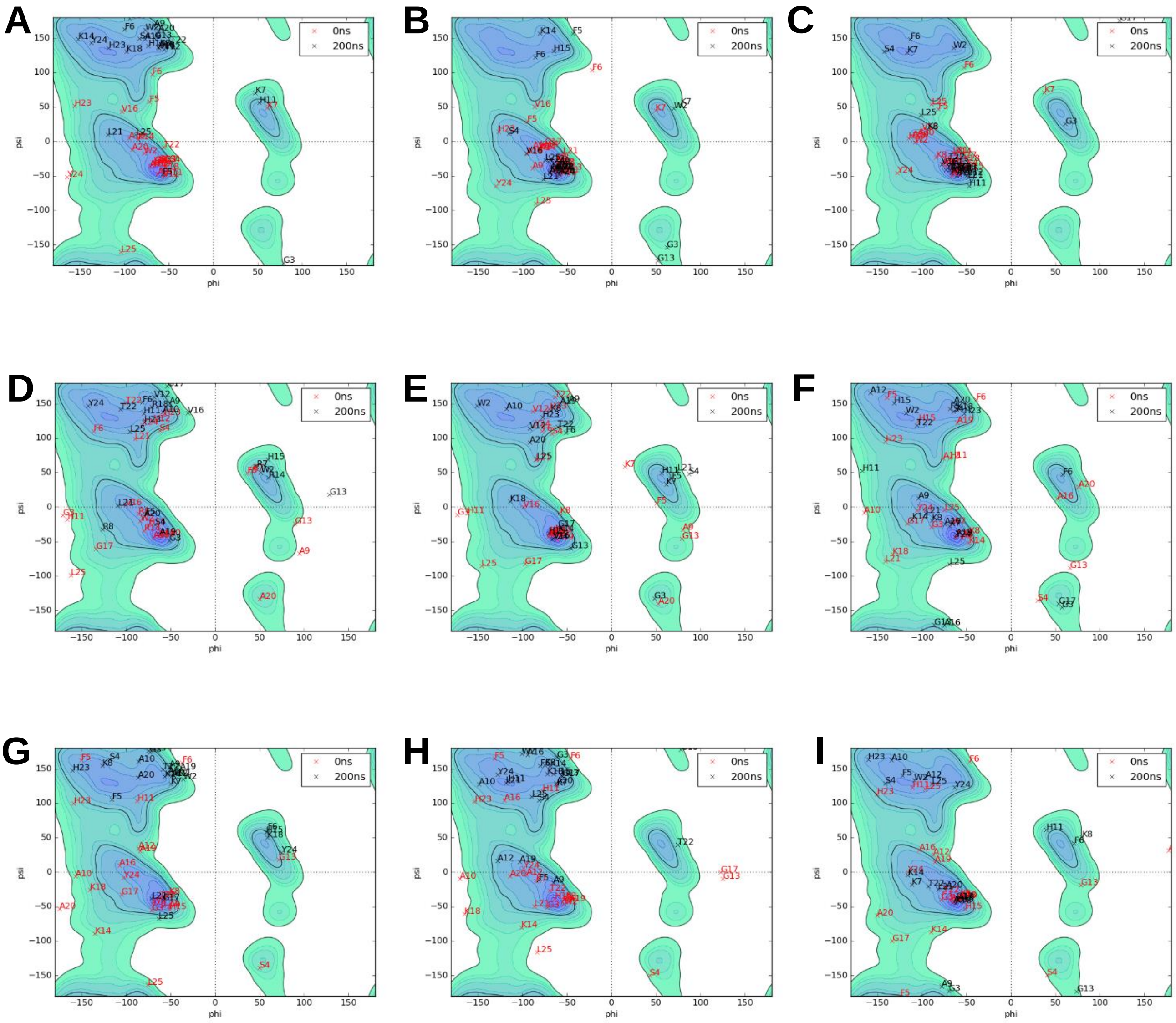


**Supplementary Figure 4. Secondary structure analysis of pleurocidin peptides from MD simulations (Ramachandran analysis).** Ramachandran plots comparing phi and psi angles for pleurocidin analogues with positively charged (A/C/D/F/G/I) or uncharged (B/E/H) histidines are shown for one representative (of four) peptide at 0 and 200 ns. Data is shown for pleurocidin (A-C), pleurocidin-KR (D-F) and pleurocidin-VA (G-I) binding to POPE/POPG (A/B/D/E/G/H) or POPG (C/F/I) bilayers.

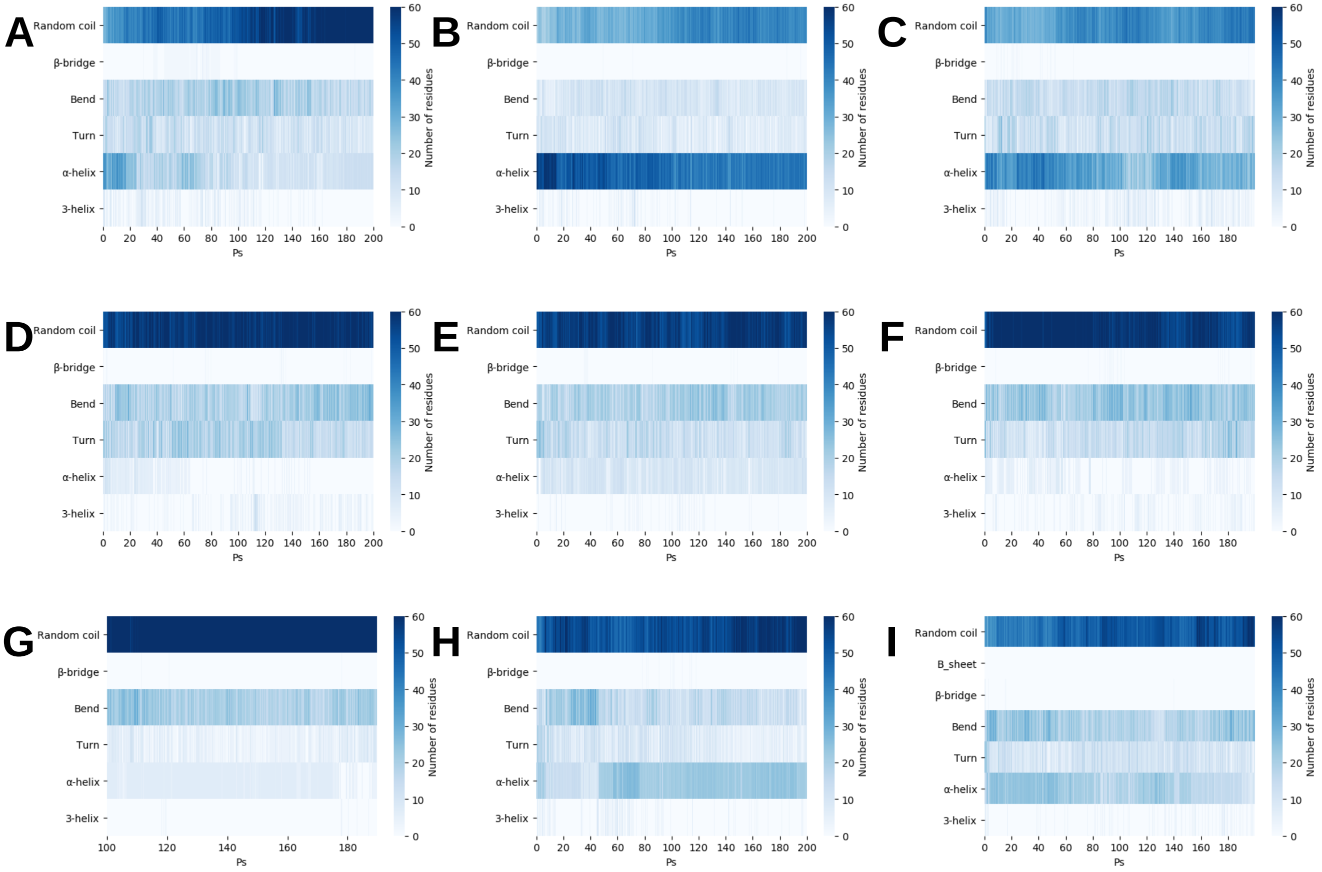


**Supplementary Figure 5. Secondary structure analysis of pleurocidin peptides from MD simulations (DSSP analysis).** Data is shown for pleurocidin analogues with positively charged (A/C/D/F/G/I) or uncharged (B/E/H) histidines binding to POPE/POPG (A/B/D/E/G/H) or POPG (C/F/I) bilayers. Data is shown for pleurocidin (A-C), pleurocidin-KR (D-F) and pleurocidin-VA (G-I). Each panel represents an average of the four peptides in each simulation.

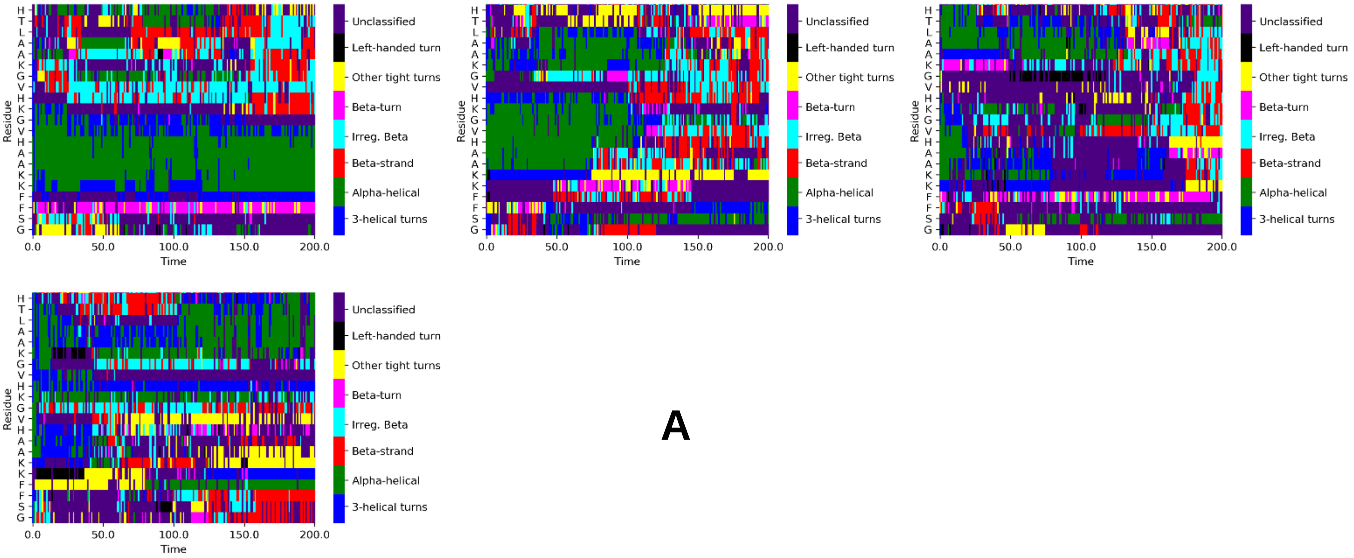


A

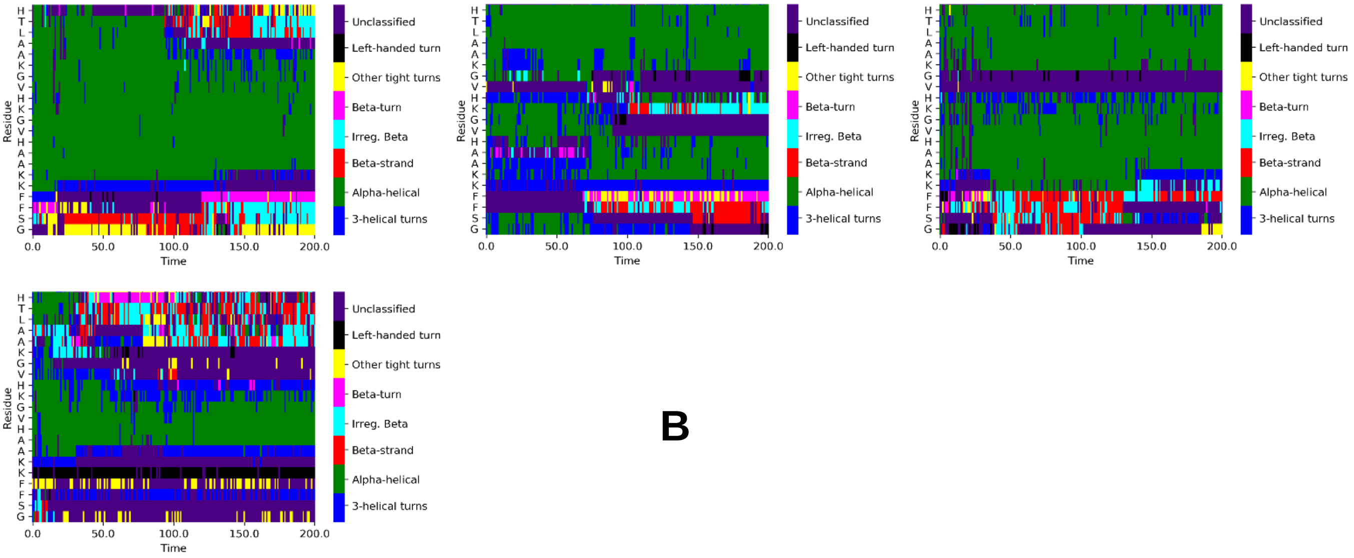


B

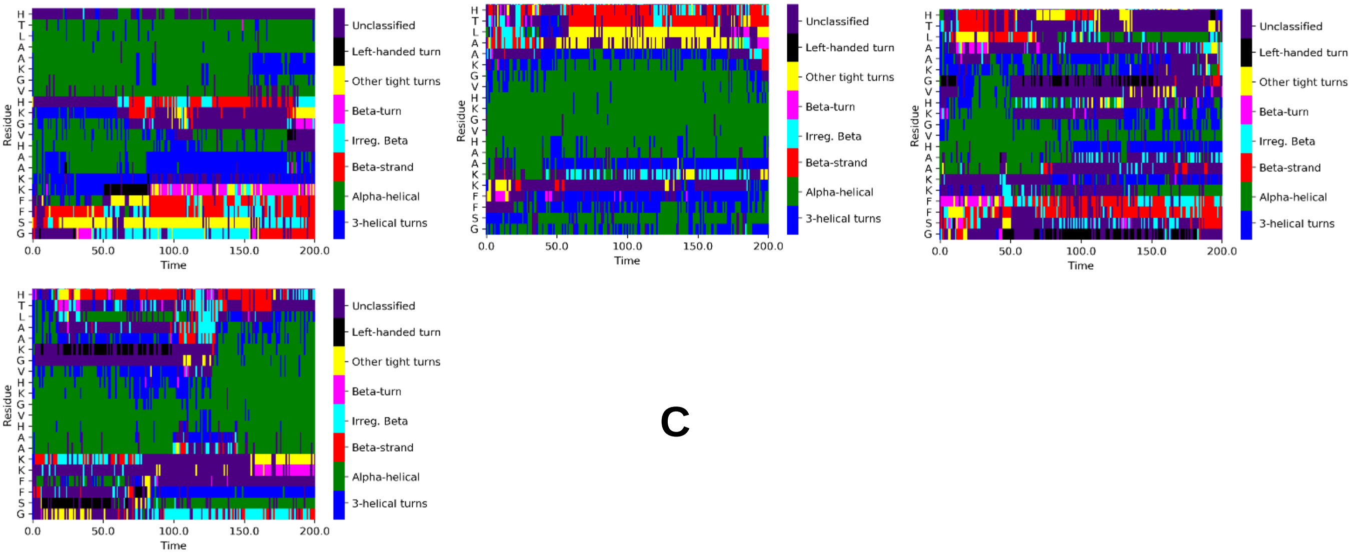


C

**Supplementary Figure 6. Secondary structure analysis of pleurocidin peptides from MD simulations (DISICL analysis).** Data is shown for pleurocidin with positively charged (A/C) or uncharged (B) histidines binding to POPE/POPG (A/B) or POPG (C) bilayers. Each panel represents one of four peptides in each simulation.

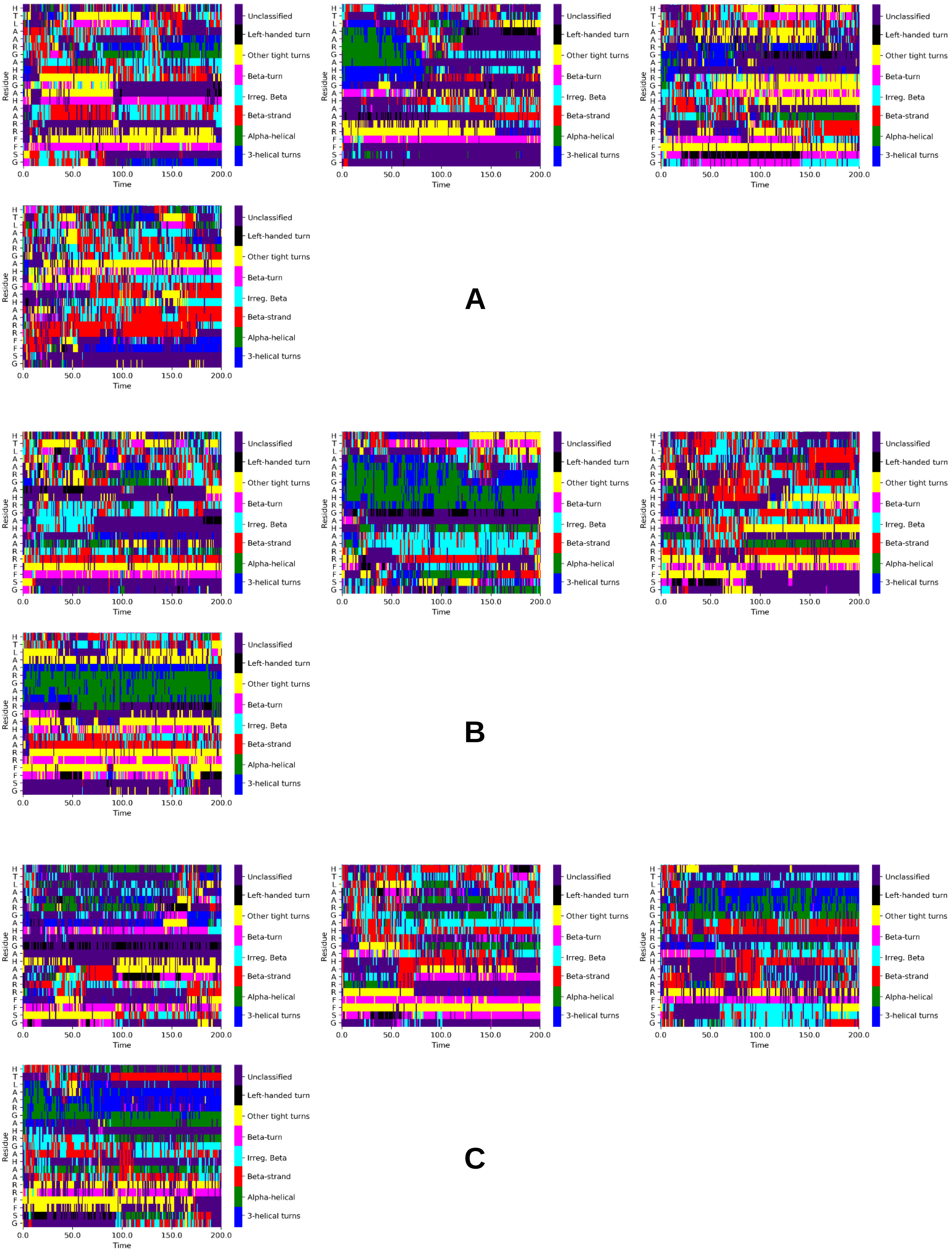


**Supplementary Figure 6 (continued). Secondary structure analysis of pleurocidin peptides from MD simulations (DISICL analysis).** Data is shown for pleurocidin-KR with positively charged (A/C) or uncharged (B) histidines binding to POPE/POPG (A/B) or POPG (C) bilayers. Each panel represents one of four peptides in each simulation.

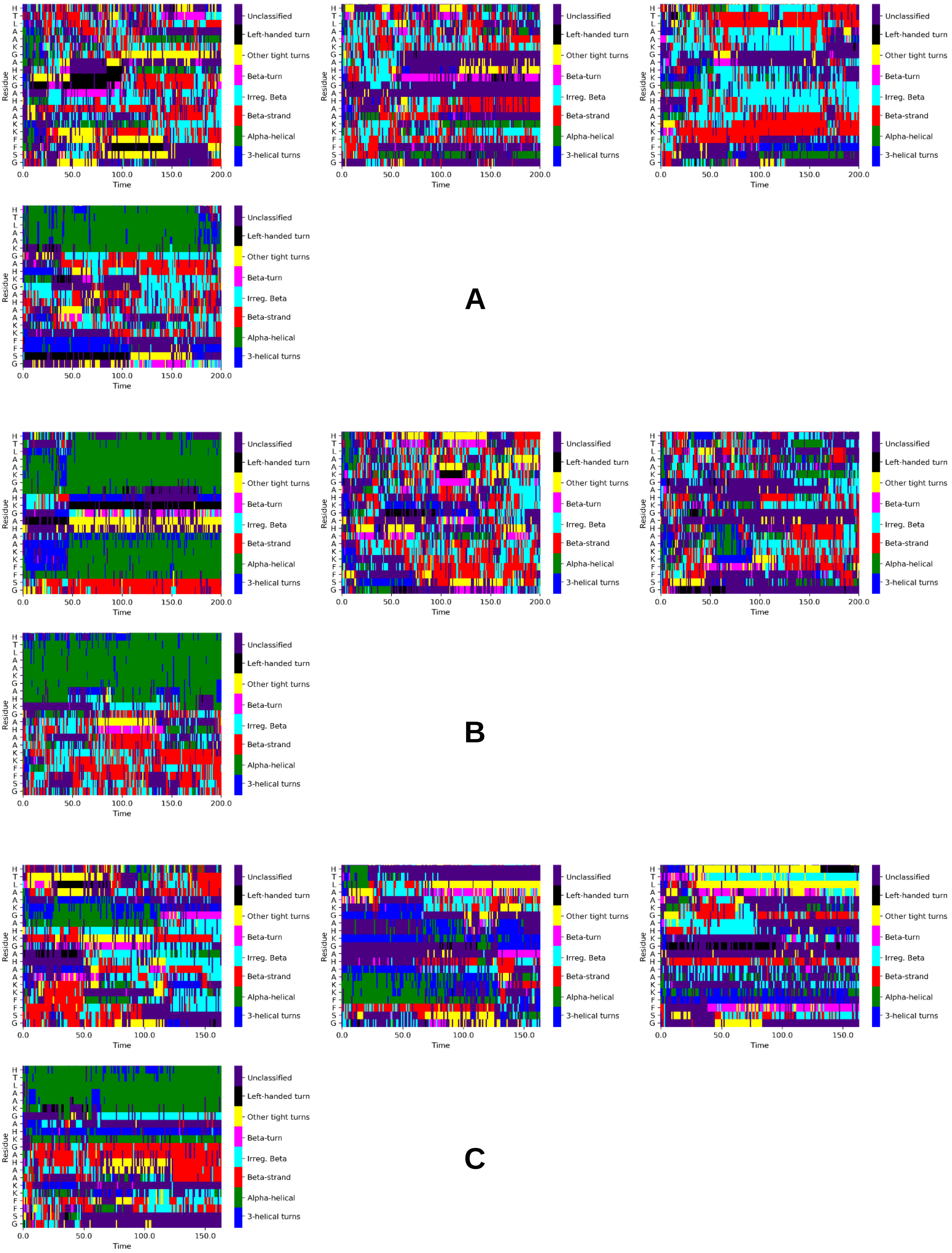


**Supplementary Figure 6 (continued) Secondary structure analysis of pleurocidin peptides from MD simulations (DISICL analysis).** Data is shown for pleurocidin-VA with positively charged (A/C) or uncharged (B) histidines binding to POPE/POPG (A/B) or POPG (C) bilayers. Each panel represents one of four peptides in each simulation.

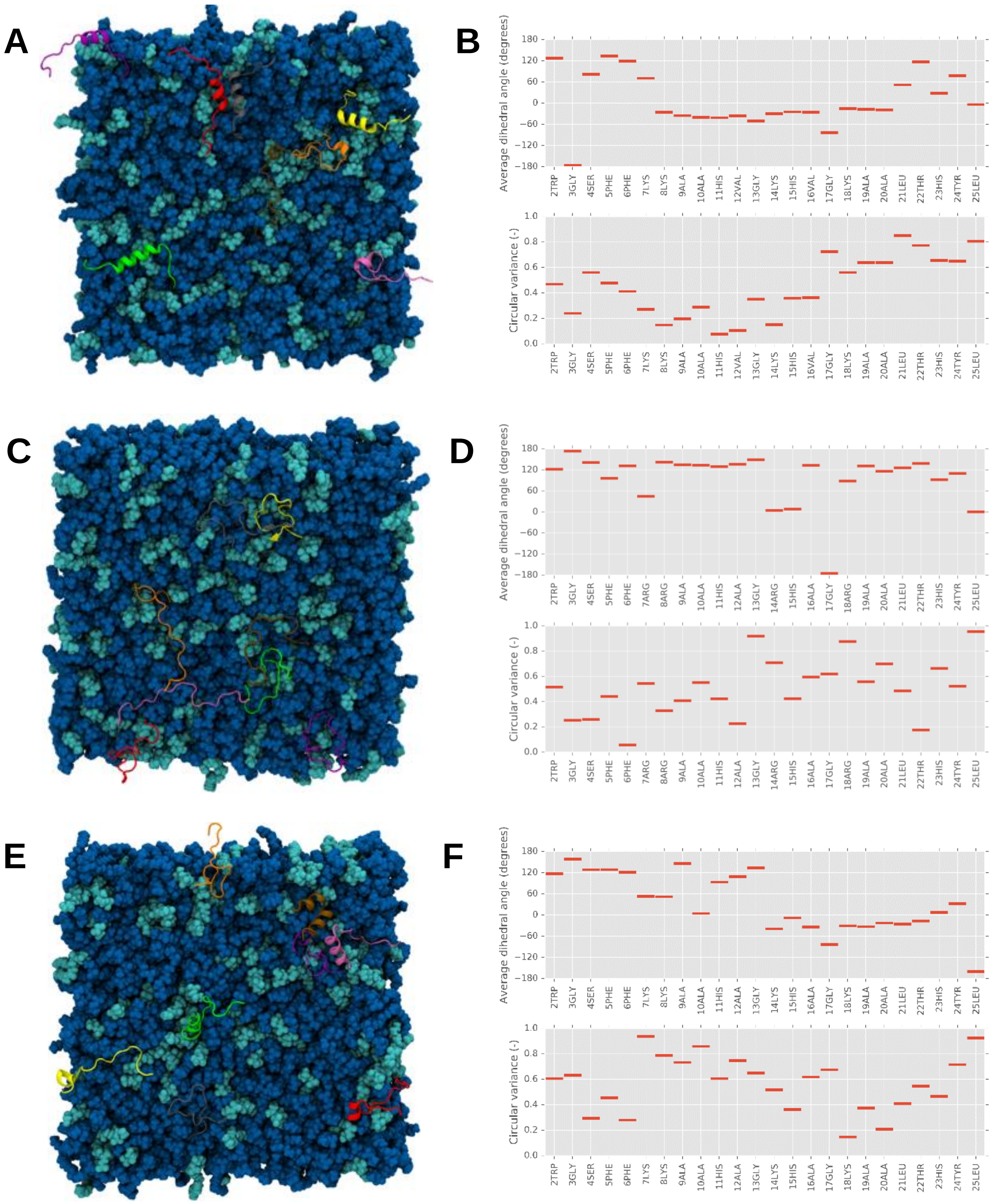


**Supplementary Figure 7. Secondary structure analysis of pleurocidin peptides from MD simulations (charged histidines, eight peptides binding to POPE/POPG bilayers).** Top view snapshots showing the tendency to aggregate and ordered/disordered conformation for each of the eight peptides (A/C/E) and the average psi dihedral angles and circular variance of psi are shown for each residue, averaged over 100 ns of simulation and eight peptides, (B/D/F) for pleurocidin (A/B), pleurocidin-KR (C/D) and pleurocidin-VA binding to POPE/POPG bilayers.

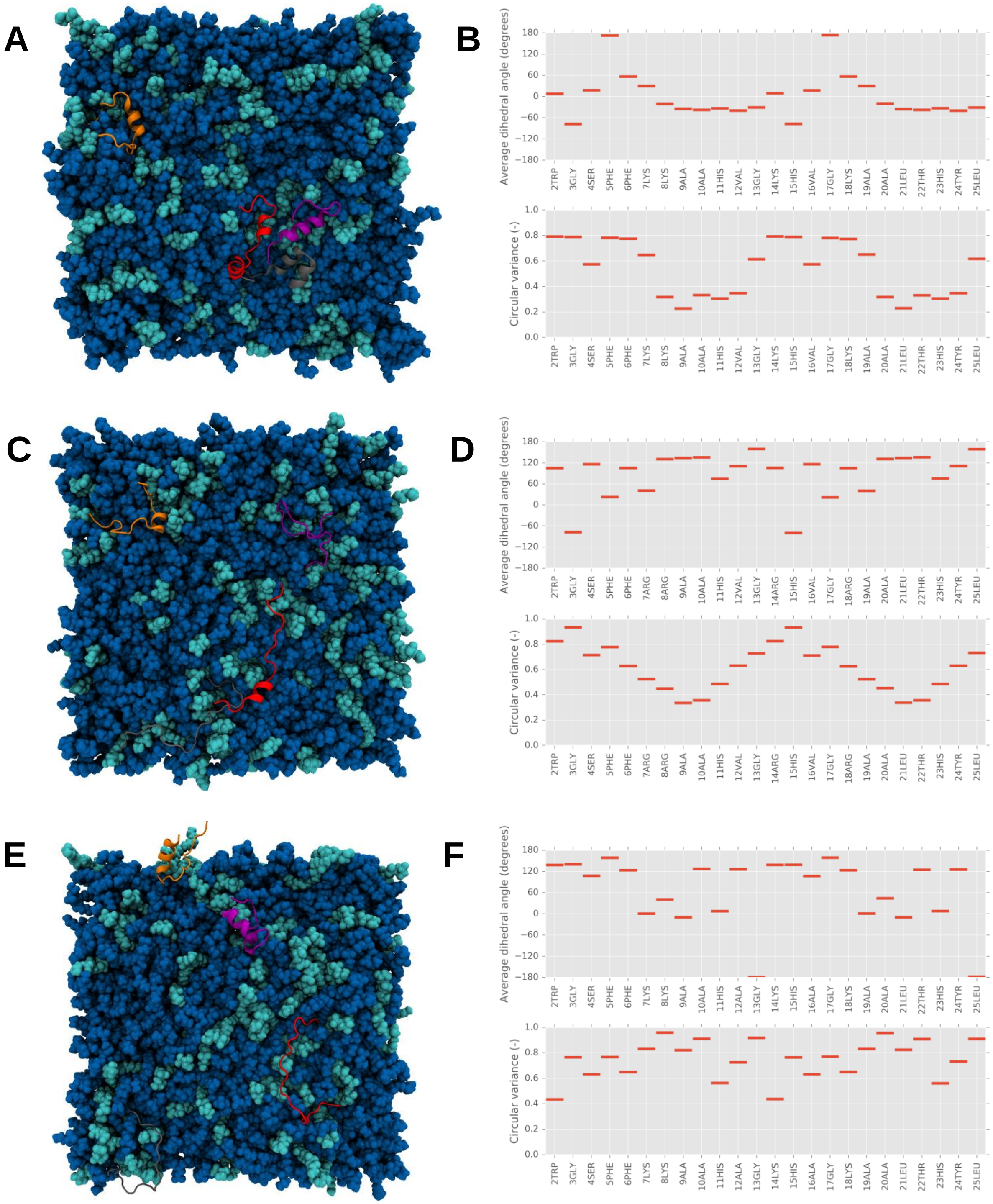


**Supplementary Figure 8. Secondary structure analysis of pleurocidin peptides from MD simulations (uncharged histidines, four peptides binding to POPE/POPG bilayers).** Top view snapshots showing the tendency to aggregate and ordered/disordered conformation for each of the four peptides (A/C/E) and the average psi dihedral angles and circular variance of psi are shown for each residue, averaged over 200 ns of simulation and four peptides, (B/D/F) for pleurocidin (A/B), pleurocidin-KR (C/D) and pleurocidin-VA binding to POPE/POPG bilayers.

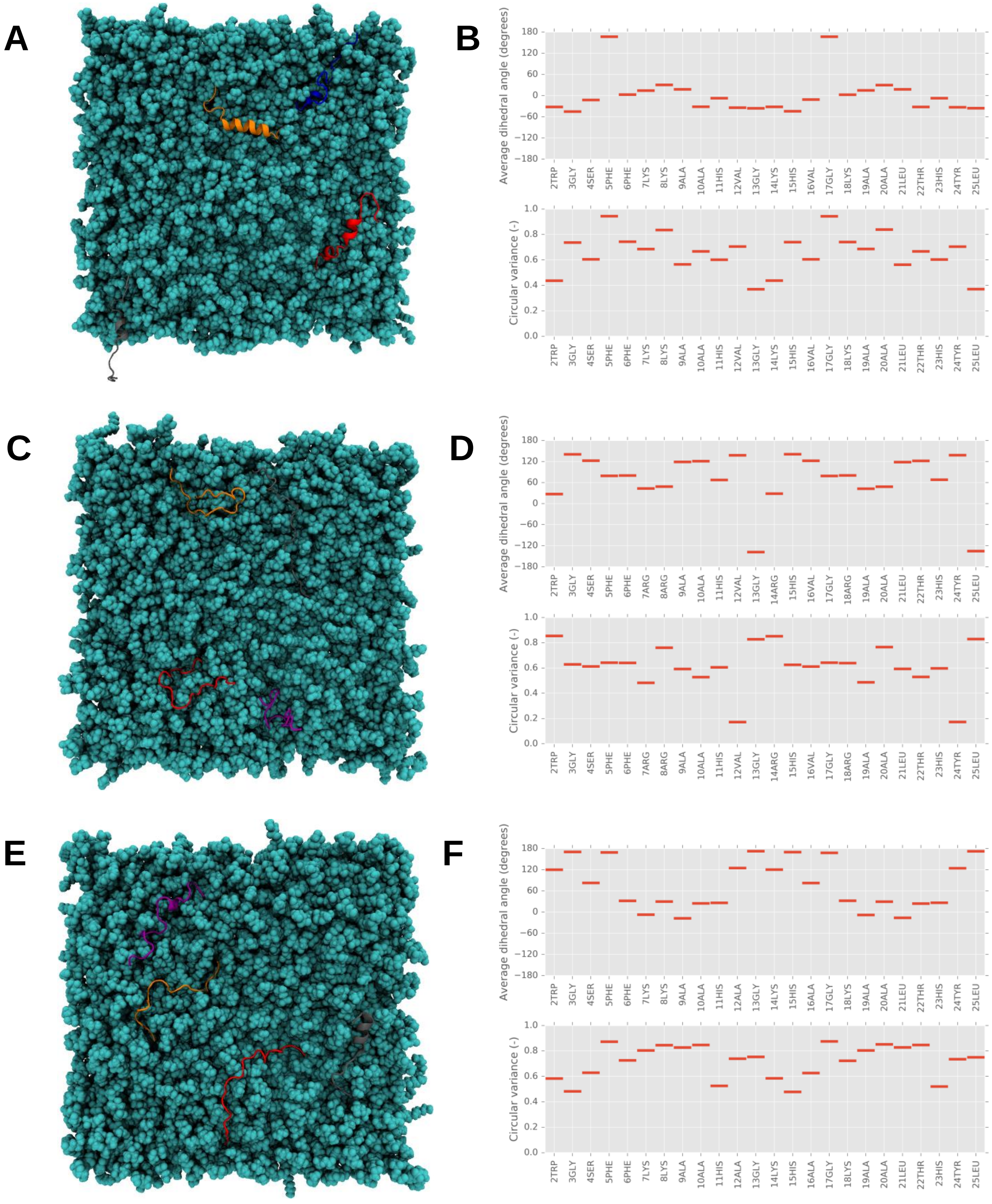


**Supplementary Figure 9. Secondary structure analysis of pleurocidin peptides from MD simulations (protonated histidines, four peptides binding to POPG bilayers).** Top view snapshots showing the tendency to aggregate and ordered/disordered conformation for each of the four peptides (A/C/E) and the average psi dihedral angles and circular variance of psi are shown for each residue, averaged over 200 ns of simulation and four peptides, (B/D/F) for pleurocidin (A/B), pleurocidin-KR (C/D) and pleurocidin-VA binding to POPG bilayers.

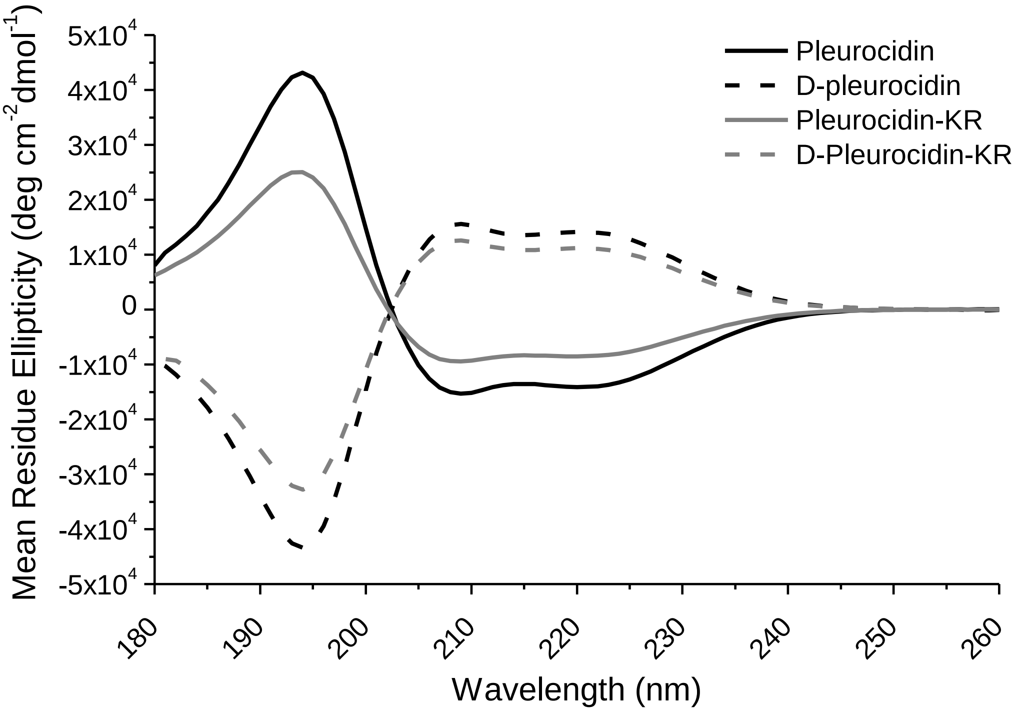


**Supplementary Figure 10. Secondary structure analysis of D- and L- amino acid enantiomers in SDS micelles.** Far-UV CD spectra are shown of the indicated peptides at 20 µM in 50 mM SDS.

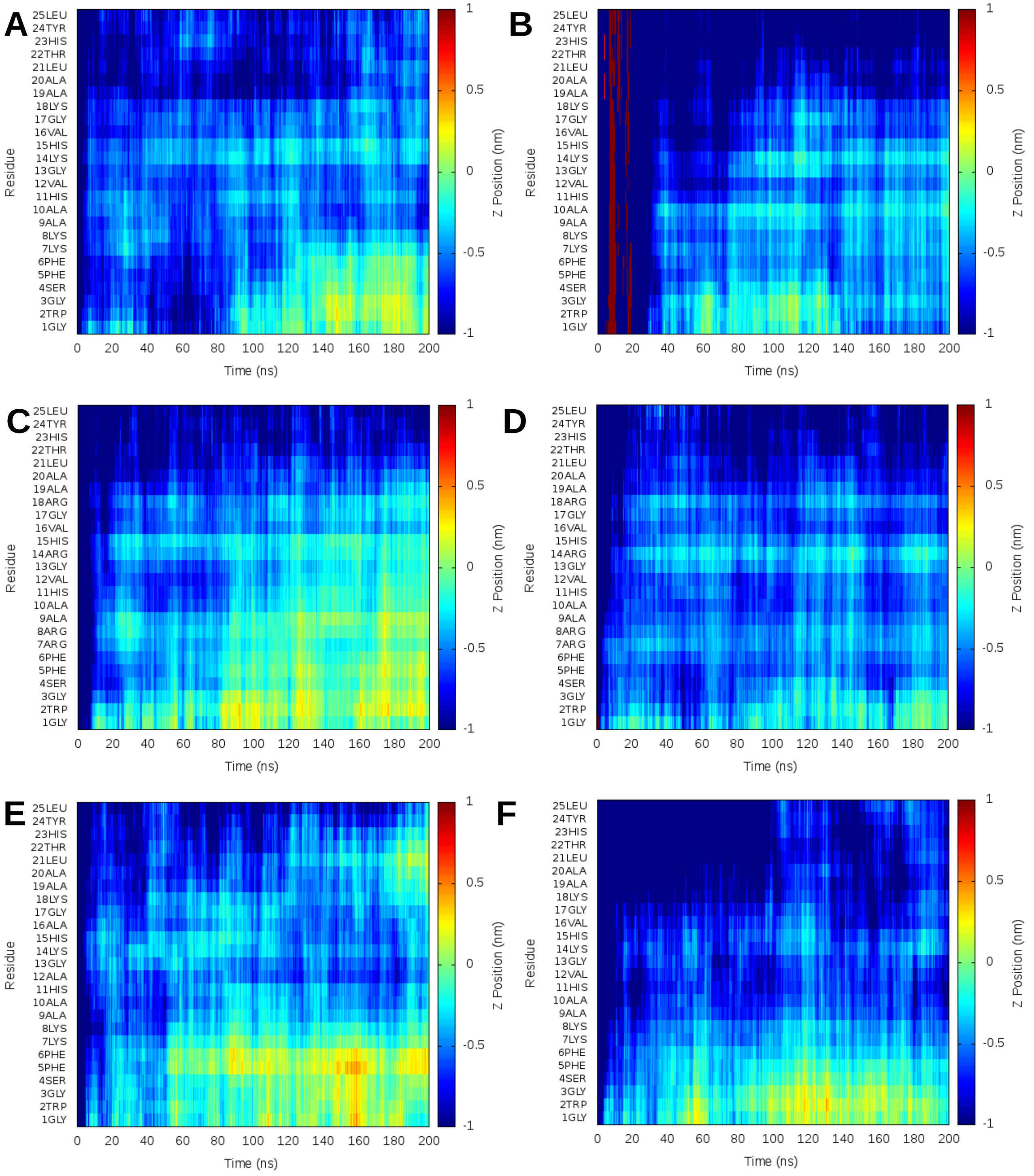


**Supplementary Figure 11. Bilayer insertion is altered in pleurocidin analogues and affected by histidine charge state.** The depth of insertion into each membrane is shown as the Z-position for each residue, averaged over all four peptides in each simulation, relative to the phosphate group plane in the upper bilayer leaflet. Positive or negative values indicate the peptides are below or above the phosphate group, for pleurocidin (A/B), pleurocidin-KR (C/D) and pleurocidin-VA binding to POPE/POPG with positively charged histidines (A/C/E) or carrying no overall charge (B/D/F). A positive charge on the three histidines is important for insertion of all three peptides but pleurocidin-VA is arguably most affected since there is little to no insertion in the C-terminal segment – consistent with the lack of peptide-lipid hydrogen bonding via Lys18 – but a much deeper insertion of the first three residues at the N-terminus.

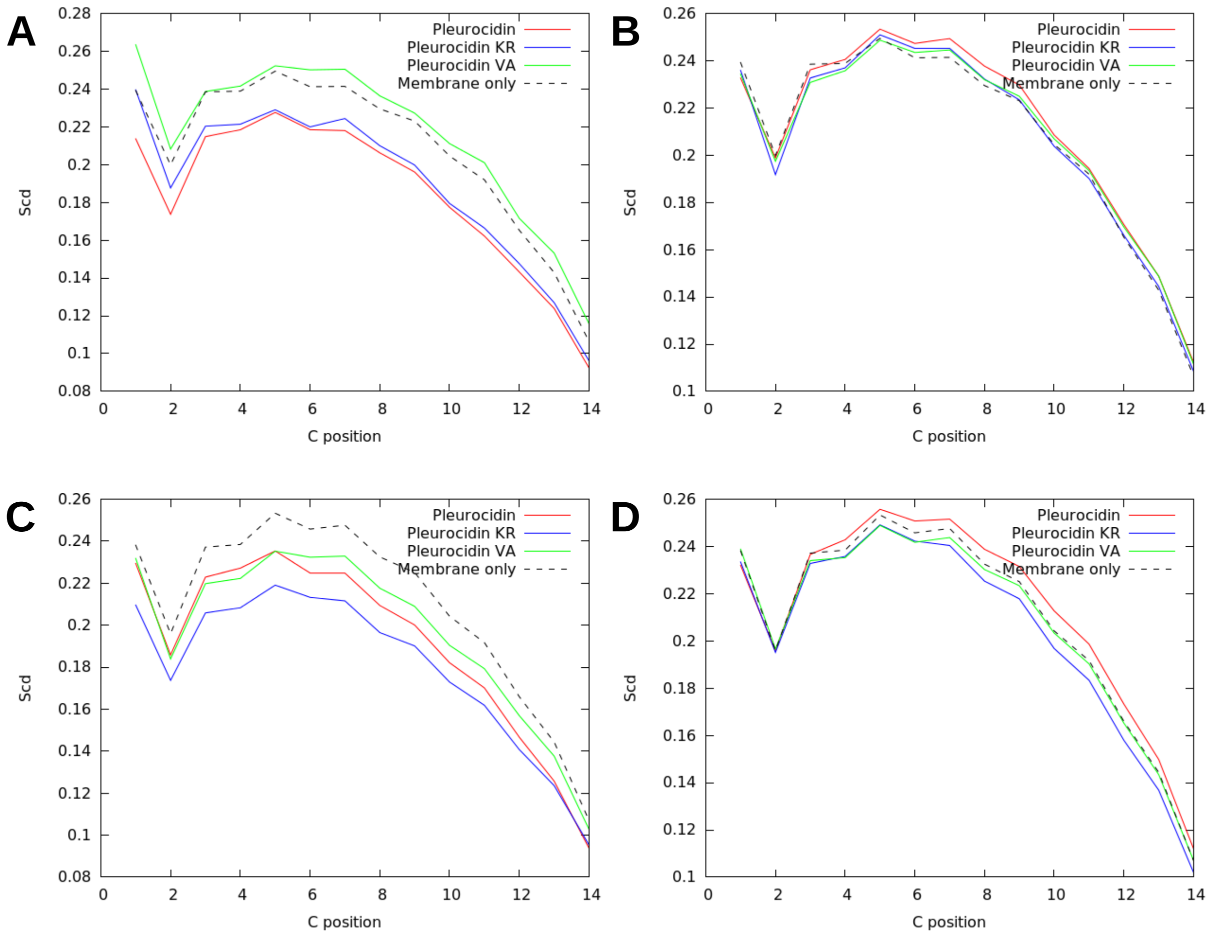


**Supplementary Figure 12. Disordering/ordering of lipid acyl chains close to or distant from each AMP.** Order parameters are shown here, averaged over the last 20 ns of the 200 ns MD simulations, for POPG (**A**/**B**) or POPE (**C**/**D**) lipid molecules within 4Å of a peptide molecule (**A**/**C**) or for the entire lipid bilayer (**B**/**D**). Lipid molecules close to a peptide may become disordered while those distant from the peptide may become more ordered. The effect on the bilayer as a whole may be a modest increase or decrease in order. MD simulation data covering the first 200 ns of a peptide-lipid interaction will not correlate perfectly with $^{2}H$ NMR data obtained in the steady-state, which, depending on time-scales, lateral diffusion and residency of any peptide annular region reports on a weighted average of lipid acyl chain order for all $^{2}H$ labelled lipids.

| | EMRSA-15 | | *P. aeruginosa* RP73 | |
|---|---|---|---|---|
| | MH | RPMI | MH | RPMI |
| D-pleurocidin / tobramycin | **0.55 ± 0.02** | 0.83 ± 0.29 | 1.00 ± 0.00 | 0.92 ± 0.14 |
| D-pleurocidin-KR / tobramycin | 0.80 ± 0.46 | 0.78 ± 0.31 | 0.85 ± 0.25 | **0.71 ± 0.07** |
| D-pleurocidin / rifampin | 1.00 ± 0.00 | 1.00 ± 0.00 | 0.85 ± 0.25 | **0.60 ± 0.04** |
| D-pleurocidin-KR / rifampin | 1.00 ± 0.00 | 1.00 ± 0.00 | 0.83 ± 0.29 | **0.61 ± 0.12** |
| D-pleurocidin / colistin | n.d | n.d | 1.00 ± 0.00 | 1.00 ± 0.00 |
| D-pleurocidin-KR / colistin | n.d. | n.d. | 0.75 ± 0.35 | 1.00 ± 0.00 |

**Supplementary Table 2. D-pleurocidin analogue synergy.** The Fractional Inhibitory Concentration (FIC) is reported for selected binary antibiotic combinations as an average ± standard error of three independent repeats.

Uncertainty regarding MIC dilution has led to a cautious adoption of FIC < 0.5 as the threshold for synergy. However, as proposed by Fratini *et* al,[54] this uncertainty can be accounted for by using reference MICs generated in the same microarray plate as the checkerboard experiment, such that more modest synergistic effects are not ignored (FIC < 1.0). Additional confidence can be gained by reporting FIC as an average ± SE.  Values shown here in bold are considered to represent synergy.

| | Isolate | Antibiotic concentration (µg/ml) | | | | |
|---|---|---|---|---|---|---|
| | | Colistin | Tobramycin | Ciprofloxacin | Gentamicin | Ceftazidime |
| Gram-negative | *Klebsiella pneumoniae* NCTC 13368 | 1 | 64 | 0.5 | 4 | 512 |
| | *Klebsiella pneumoniae* M6 | 2 | 8 | <0.5 | 0.25 | <0.5 |
| | *Acinetobacter baumannii* AYE | 1 | 64 | 128 | >512 | >128 |
| | *Acinetobacter baumannii* ATCC 17978 | 1 | 4 | 0.5 | 0.5 | <0.5 |
| | *Pseudomonas aeruginosa* PAO1 | 2 | 2 | 0.5 | 4 | 1 |
| | *Pseudomonas aeruginosa* NCTC 13437 | 4 | 64 | 64 | 256 | >128 |
| | *Escherichia coli* NCTC 12923 | 0.0625 | 16 | ≤0.125 | 1 | ≤0.125 |
| Gram-positive | MS *Staphylococcus aureus* ATCC 9144 | ND | 0.25-0.5 | 1 | 0.25 | 16 |
| | EMR *Staphylococcus aureus*-15 NCTC 13616 | >128 | 0.5 | 64 | 0.25 | 128 |
| | EMR *Staphylococcus aureus*-16 NCTC 13277 | ND | 0.25 | 128 | 0.25 | 8 |
| | VS *Enterococcus faecalis* NCTC 775 | ND | 4 | 2 | 4 | 32 |
| | VR *Enterococcus faecium* NCTC 12204 | ND | 64 | 1 | 4 | >128 |

**Supplementary Table 3. Antibiogram for Gram-negative and Gram-positive bacteria employed in the AMP susceptibility testing**

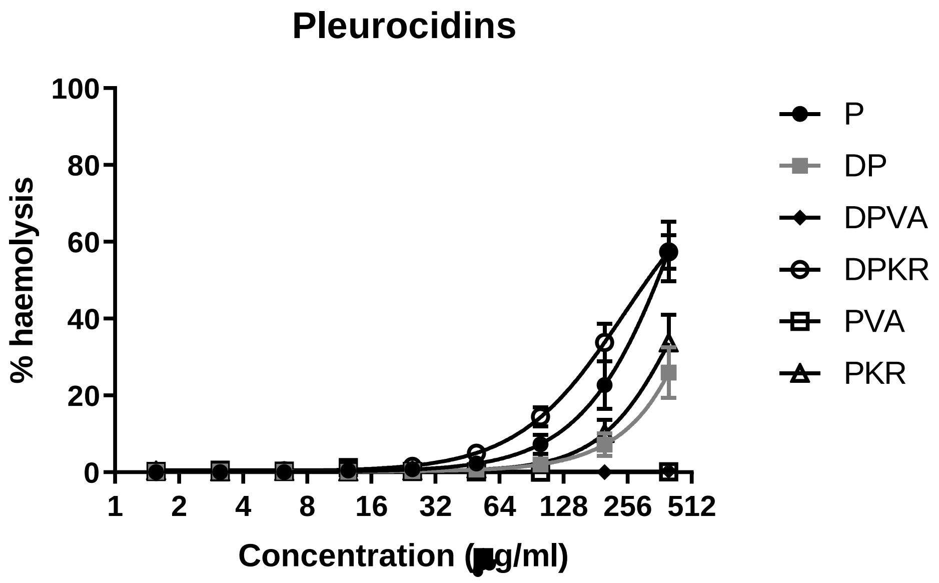


**Supplementary Figure 13. Pleurocidin, D-pleurocidin and their analogues all have weak haemolytic potential.** None of the peptides cause significant haemolysis close to the MIC i.e. below 4 µg/ml. 10% haemolysis is achieved with ~ 75 µg/ml with D-pleurocidin-KR (most haemolytic), 120 µg/ml pleurocidin, 192 µg/ml pleurocidin-KR, 240 µg/ml D-pleurocidin. Only pleurocidin and D-pleurocidin-KR attain 50% haemolysis at the highest concentration tested 400 µg/ml. Neither pleurocidin-VA nor D-pleurocidin-VA induce detectable haemolysis, even at the highest concentration tested.

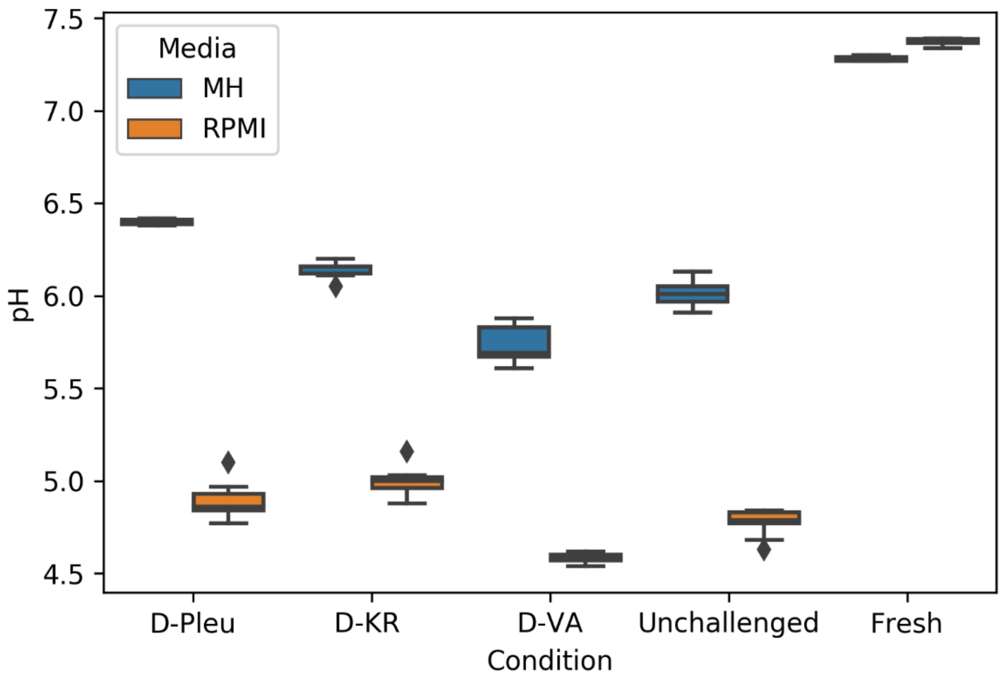


**Supplementary Figure 14. The metabolic strategy and effect of each peptide is reflected in the spent culture pH after EMRSA-15 growth.** EMRSA-15 uses fermentative pathways in both media, producing lactate ethanol and, in MHB, formate. However much more glucose is available in RPMI than in MHB and hence much more lactate is produced, and this is associated with a much greater acidification of the spent media. Fermentation is halted in MHB when EMRSA-15 is challenged with D-pleurocidin but not the two analogues. In RPMI fermentation continues irrespective of the challenge conditions.

The box shows the quartiles of the dataset while the whiskers extend to the lowest or highest data point still within 1.5 inter quartile range of the lower or upper quartile.

| | HR-MAS | | | Liquid-state | | |
|---|---|---|---|---|---|---|
| | Model | $Q^2$ | Permutated $Q^2$ | Model | $Q^2$ | Permutated $Q^2$ |
| *S. aureus* EMRSA-15 | Unchallenged v D-pleurocidin-KR MHB | 0.844 | -0.221 | MHB v D-pleurocidin-KR | 0.962 | -0.362 |
| | Unchallenged v D-pleurocidin MHB | 0.805 | -0.221 | MHB v D-pleurocidin | 0.988 | -0.278 |
| | Unchallenged v D-pleurocidin-VA MHB | 0.925 | -0.204 | MHB v D-pleurocidin-VA | 0.998 | -0.386 |
| | Unchallenged v D-pleurocidin-KR RPMI | 0.874 | -0.242 | RPMI v D-pleurocidin-KR | 0.987 | -0.211 |
| | Unchallenged v D-pleurocidin RPMI | 0.685 | -0.298 | RPMI v D-pleurocidin | 0.989 | -0.206 |
| | D-pleurocidin-VA RPMI | 0.899 | -0.216 | RPMI v D-pleurocidin-VA | 0.992 | -0.242 |
| | - | - | - | Fresh MHB v unchallenged | 0.996 | -0.339 |
| | - | - | - | Fresh RPMI v unchallenged | 0.988 | -0.252 |
| *P. aeruginosa* RP73 | Unchallenged v D-pleurocidin MHB | 0.447 | -0.280 | MHB v D-pleurocidin | 0.985 | -0.308 |
| | Unchallenged v D-pleurocidin-KR MHB | 0.766 | -0.252 | MHB v D-pleurocidin-KR | 0.985 | -0.235 |
| | Unchallenged v tobramycin MHB | 0.516 | -0.223 | MHB v tobramycin | 0.983 | -0.279 |
| | Unchallenged v D-pleurocidin RPMI | 0.983 | -0.172 | RPMI v D-pleurocidin | 0.991 | -0.145 |
| | Unchallenged v D-pleurocidin-KR RPMI | 0.983 | -0.172 | RPMI v D-pleurocidin-KR | 0.985 | -0.123 |
| | Unchallenged v tobramycin RPMI | 0.969 | -0.163 | RPMI v tobramycin | 0.989 | -0.100 |
| | - | - | - | Fresh MHB v unchallenged | 0.991 | -0.262 |
| | - | - | - | Fresh RPMI v unchallenged | 0.988 | -0.102 |

**Supplementary Table 4. $Q^2$ and permutated $Q^2$ for OPLS-DA models for the key comparisons described in the study.** In all cases good models were achieved and in most cases these were excellent. Notably this indicates that the effect of each sub-inhibitory antibiotic challenge was significant.

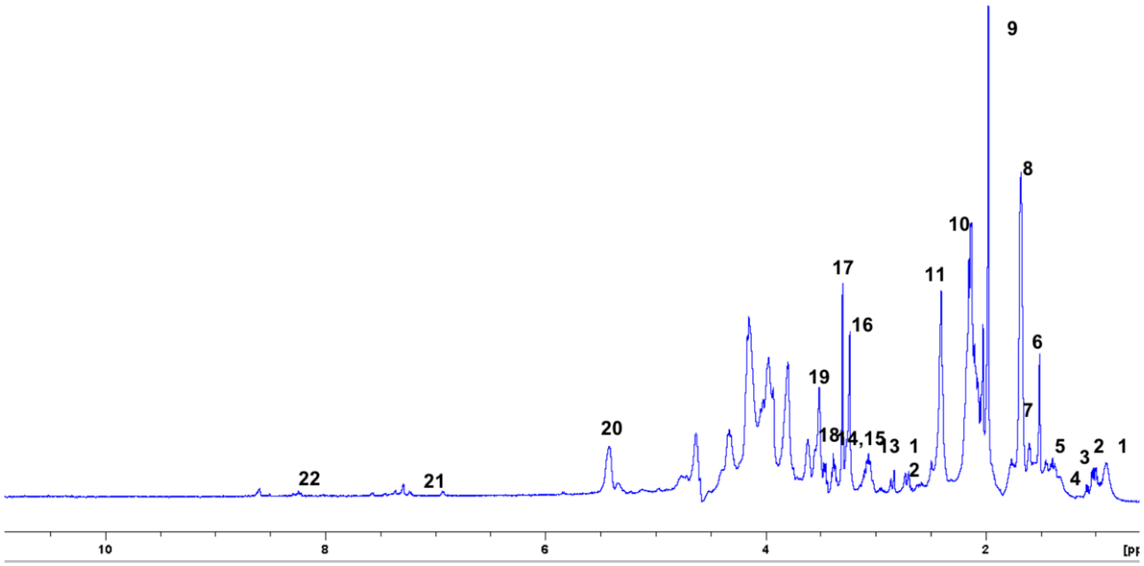


**Supplementary Figure 15A. Representative metabolite assignments on 1D NMR spectrum for EMRSA-15 intracellular metabolites.** Spectrum of EMRSA-15 collected using $^1$H HR-MAS NMR. Only assignable metabolites are shown: (1) lipid-$CH_3$, (2) 2-aminobutyrate, (3) alloisoleucine, (4) valine, (5) lipid-$CH_2$ (6) alanine, (7) D-alanine, (8) putrescine, (9) acetate, (10) N-acetyl-x, (11) glutamate, (12) succinate, (13) aspartate, (14) ornithine, (15) ethanolamine, (16) choline, (17) betaine, (18) proline, (19) glycerol (lipid headgroup) (t), (20) lipid -CH=CH-, (21) uracil, (22) adenosine.

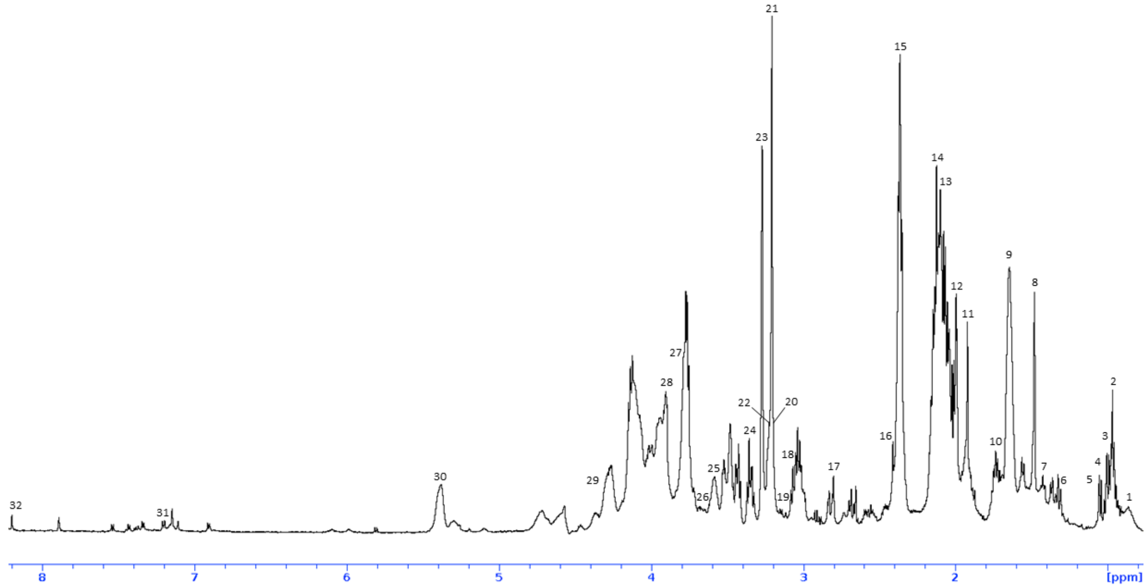


**Supplementary Figure 15B. Representative metabolite assignments on 1D NMR spectrum for *P. aeruginosa* RP73 intracellular metabolites.** Spectrum of *P. aeruginosa* RP73 collected using $^1$H HR-MAS NMR assignments are: (1) lipid $CH_3$ groups, (2) leucine, (3) 2-aminobutyrate, (4) isoleucine, (5) valine, (6) lactate, (7) alanine, (8) unassigned, (9) D-alanine (lipoteichoic acid), (10) lysine, (11) acetate, (12) N-acetyl-x(1), (13) N-acetyl-x(2), (14) N-acetyl-x(3), (15) glutamate, (16) succinate, (17) unassigned, (18) ornithine, (19) citrulline, (20) choline, (21) acetyl/phosphocholine, (22) acetyl/phosphocholine, (23) betaine, (24) proline, (25) unassigned, (26) glycerol (lipid headgroup 1), (27) glycerol (lipid headgroup 2), (28) glycerol (lipid headgroup 3), (29) unassigned, (30) unassigned, (31) uracil, (32) adenosine.

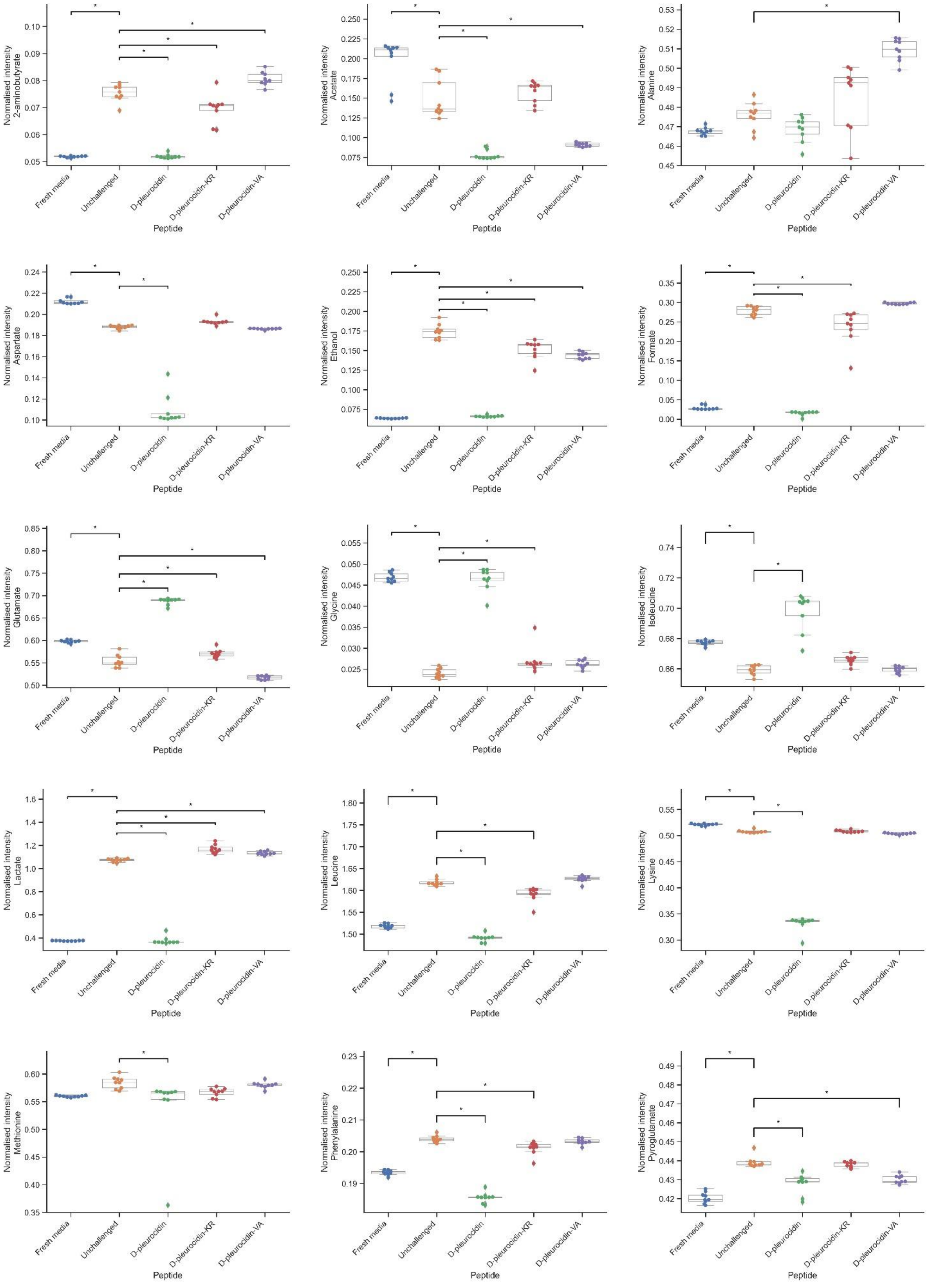


**Supplementary Figure 16. Substantial differences in the metabolic strategy of EMRSA-15 following challenge by D-pleurocidin analogues in MHB.** Univariate analysis of individual metabolite variation as observed by $^1$H NMR of Mueller-Hinton broth with EMRSA-15 cultured and challenged as indicated.
For this and subsequent plots, the box shows the quartiles of the dataset while the whiskers extend to the lowest or highest data point still within 1.5 inter quartile range of the lower or upper quartile.

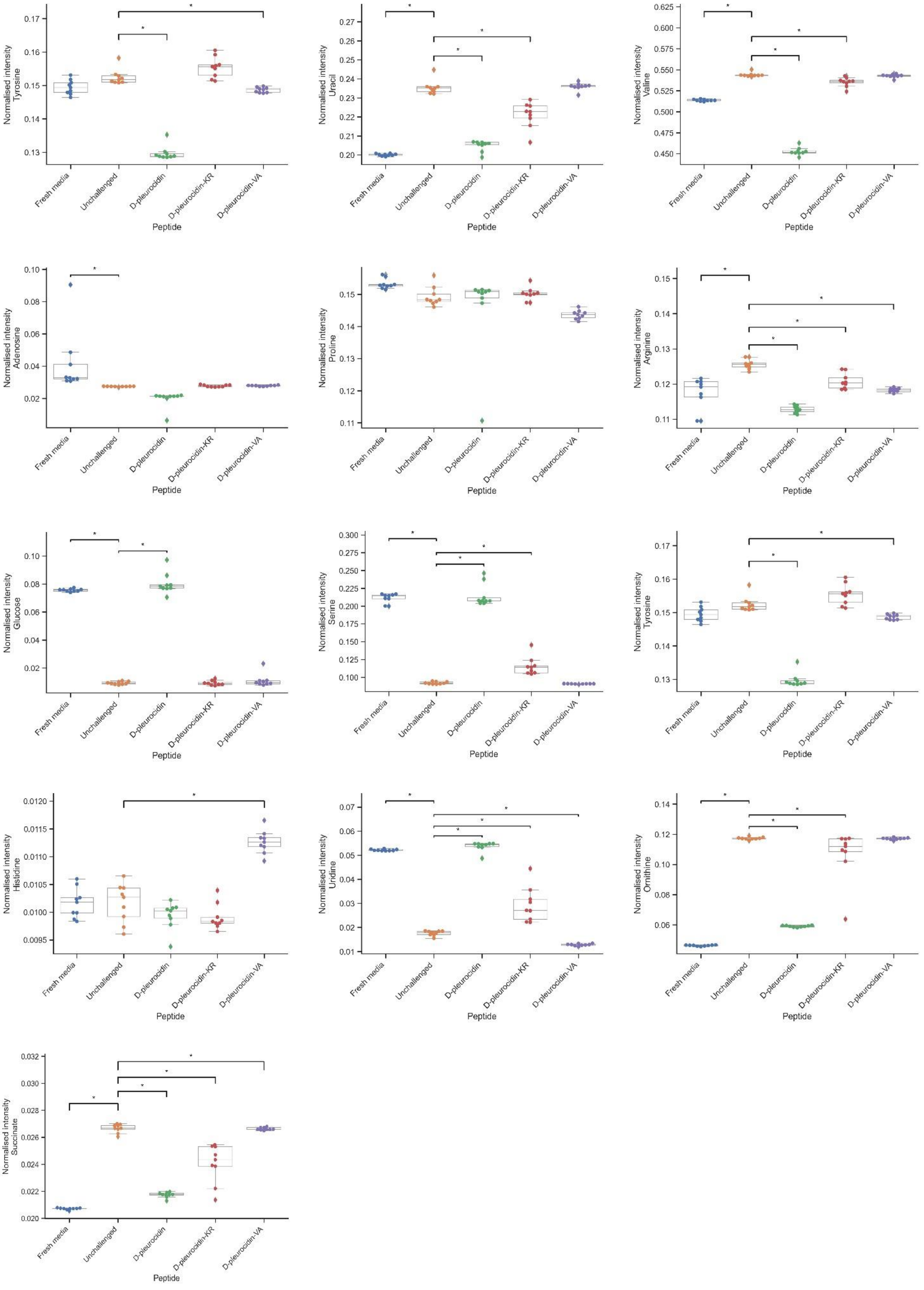


**Supplementary Figure 16 (continued). Substantial differences in the metabolic strategy of EMRSA-15 following challenge by D-pleurocidin analogues in MHB.** Univariate analysis of individual metabolite variation as observed by $^{1}$H NMR of Mueller-Hinton broth with EMRSA-15 cultured and challenged as indicated.

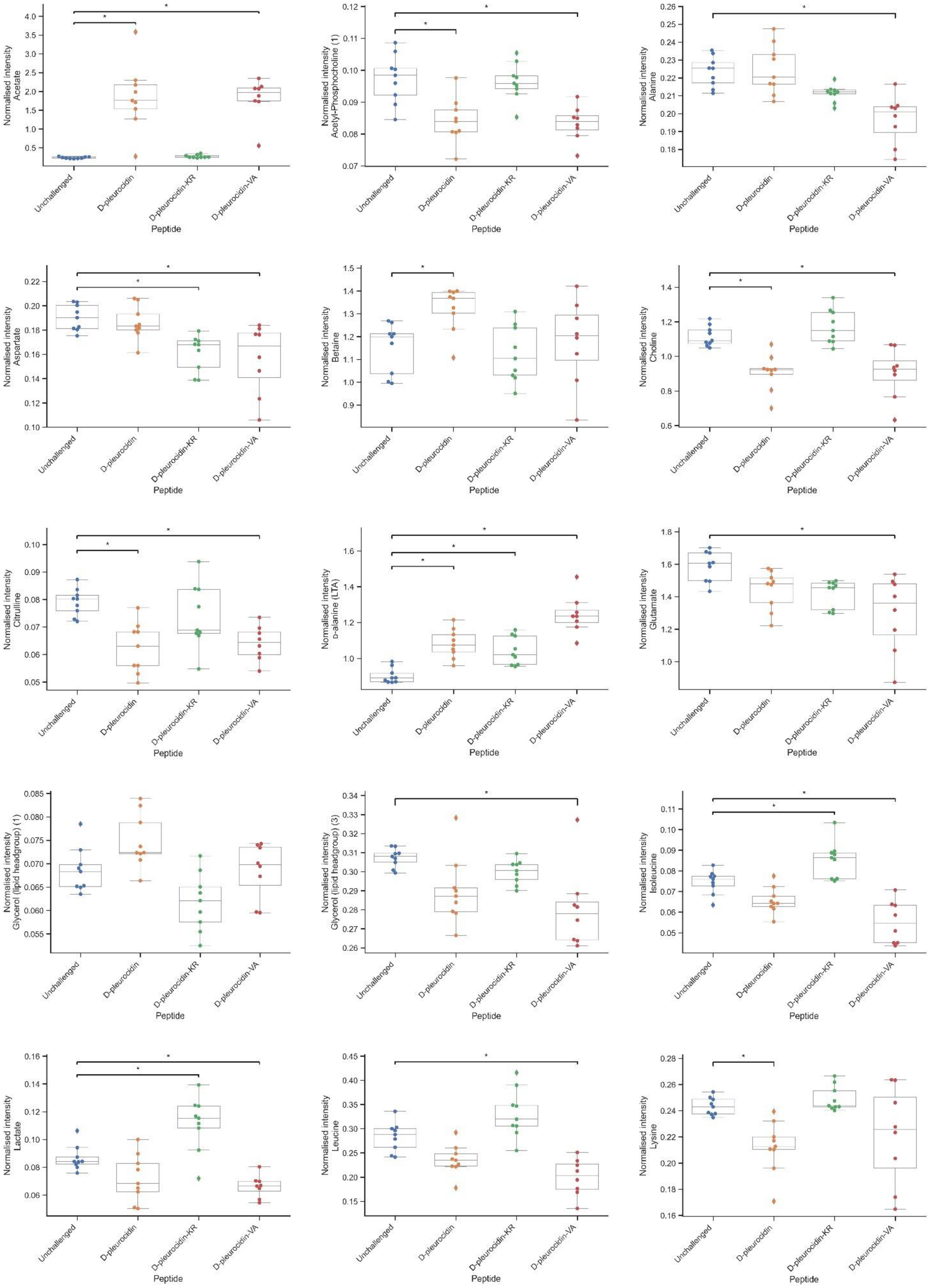


**Supplementary Figure 17. Substantial differences in the response of EMRSA-15 to challenge by pleurocidin analogues in MHB.** Univariate analysis of individual cellular metabolite variation as observed by $^{1}$H HR-MAS NMR of EMRSA-15 cultured in Mueller-Hinton broth and challenged as indicated.

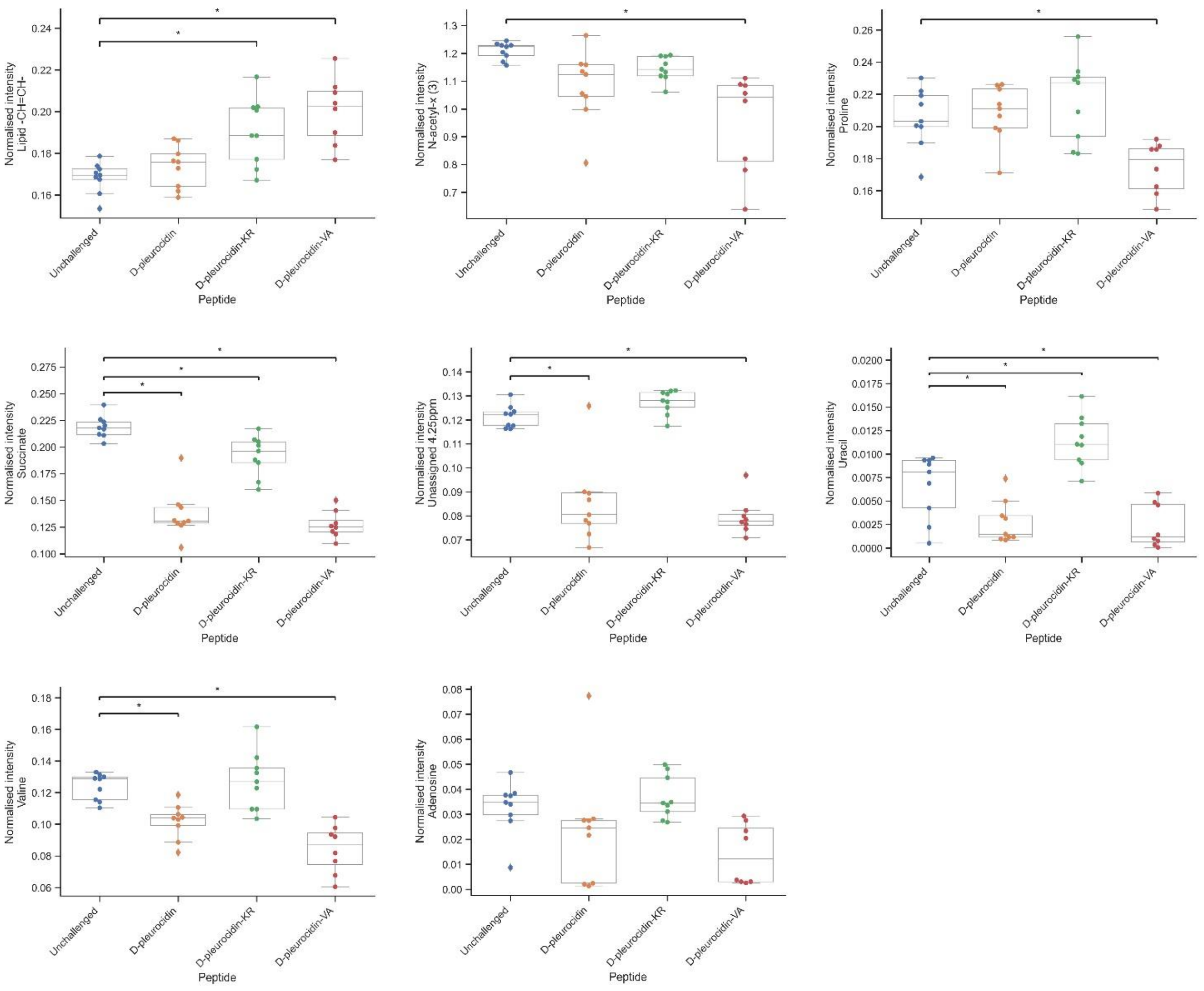


**Supplementary Figure 17 (continued). Substantial differences in the response of EMRSA-15 to challenge by pleurocidin analogues in MHB.** Univariate analysis of individual cellular metabolite variation as observed by $^1$H HR-MAS NMR of EMRSA-15 cultured in Mueller-Hinton broth and challenged as indicated. Other metabolites detected but where there were no significant changes or trends, relative to unchallenged bacteria, include 2-aminobutyrate, glycerol lipid headgroup (2), lipid $CH_3$, two N-acetyl-x resonances and unassigned resonances at 1.47, 3.48, and 8.55 ppm.

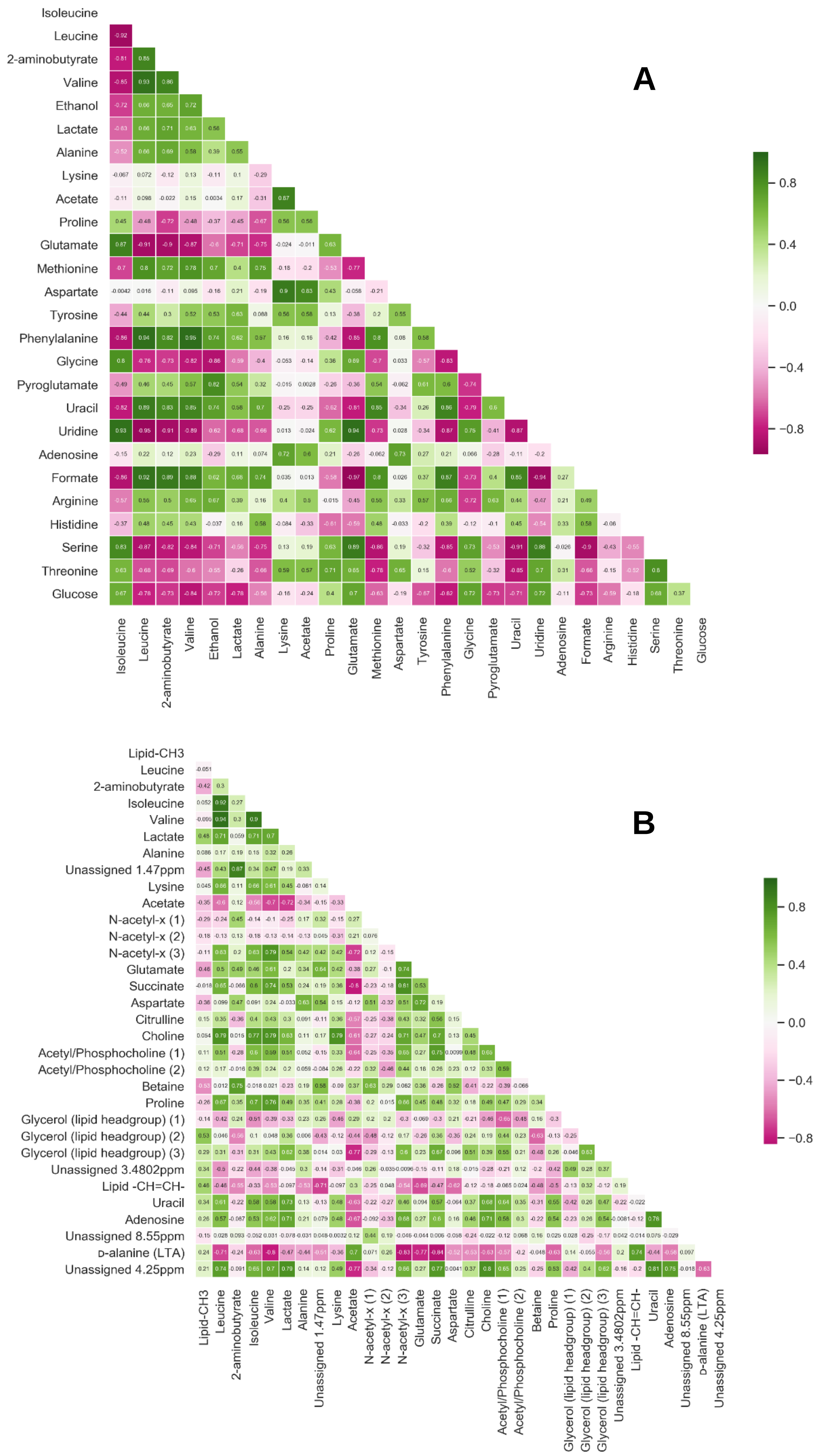


**Supplementary Figure 18. Spearman correlations between metabolites – EMRSA-15 in MHB.** Spearman correlation matrices are shown for spent media (A) and cellular (B) metabolites across all samples where EMRSA-15 is cultured in MHB (includes presence/absence of each of three D-pleurocidin analogues).

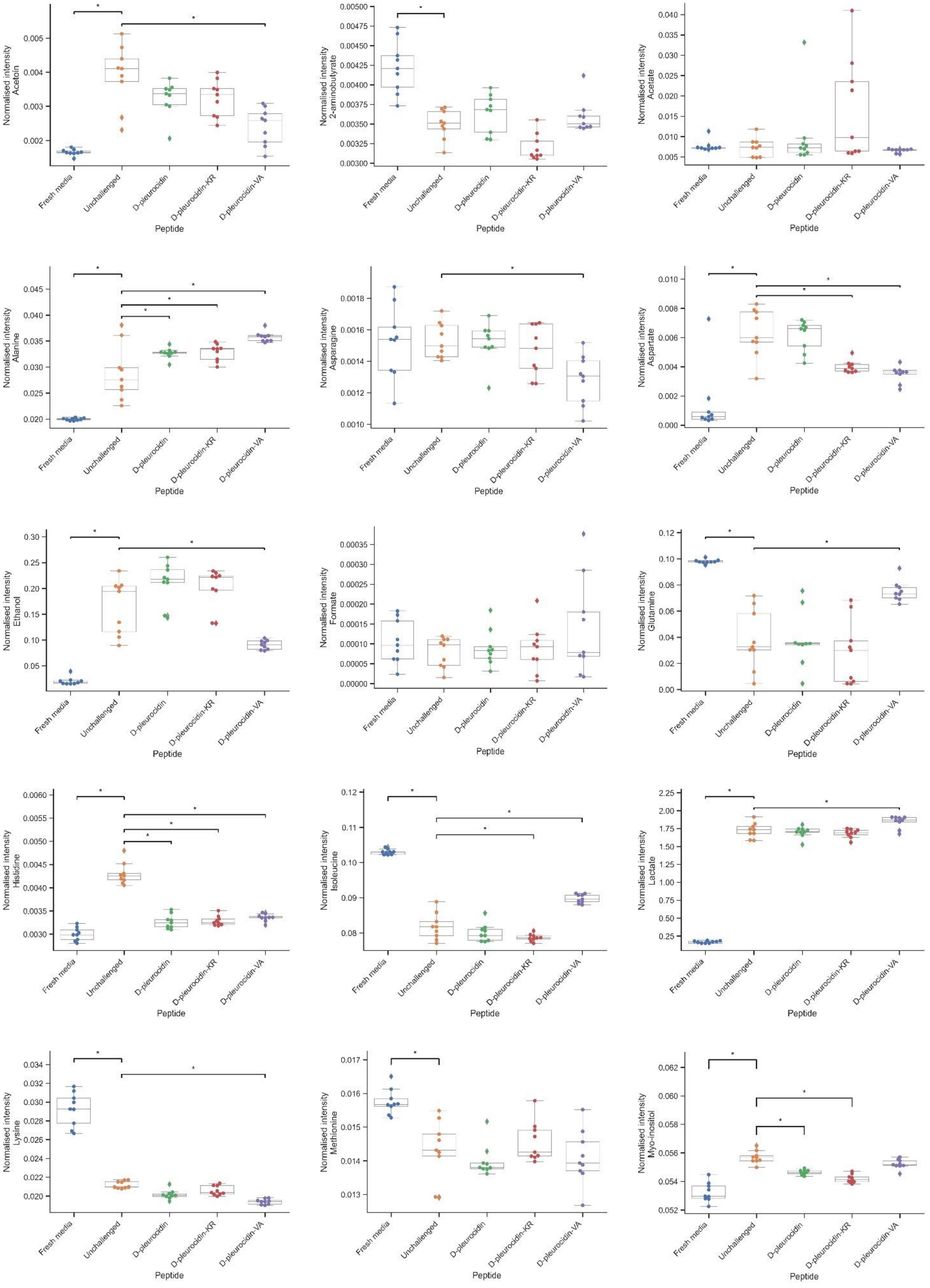


**Supplementary Figure 19. Modest differences in the metabolic strategy of EMRSA-15 following challenge by D-pleurocidin analogues in RPMI.** Univariate analysis of individual metabolite variation as observed by $^{1}H$ NMR of RPMI with EMRSA-15 cultured and challenged as indicated.

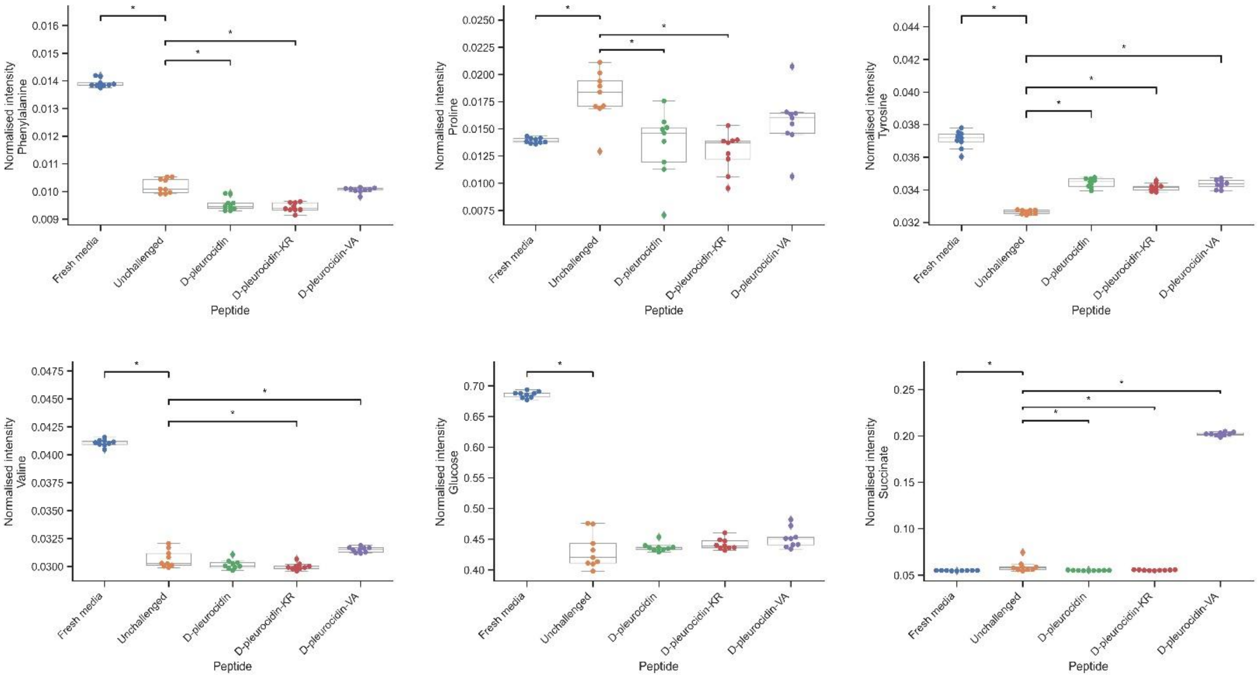


**Supplementary Figure 19 (continued). Modest differences in the metabolic strategy of EMRSA-15 following challenge by D-pleurocidin analogues in RPMI.** Univariate analysis of individual metabolite variation as observed by $^{1}H$ NMR of RPMI with EMRSA-15 cultured and challenged as indicated.

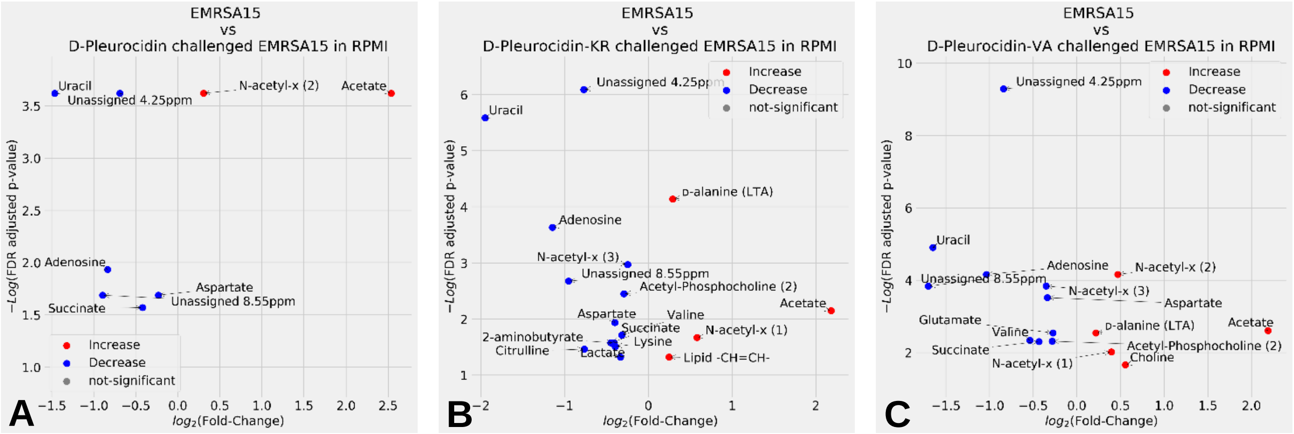


**Supplementary Figure 20. Modest differences in the response of EMRSA-15 to challenge by pleurocidin analogues in RPMI.** Volcano plots obtained from $^{1}H$ HR-MAS NMR of bacterial pellets reveals the effect on cellular metabolites of growth challenge with the indicated antibiotics. Data is shown for EMRSA-15 cultured in RPMI (5% FBS) and challenged with D-pleurocidin (A), D-pleurocidin-KR (B) or D-pleurocidin-VA (C). Metabolites with non-significant changes are not shown to avoid overlap.

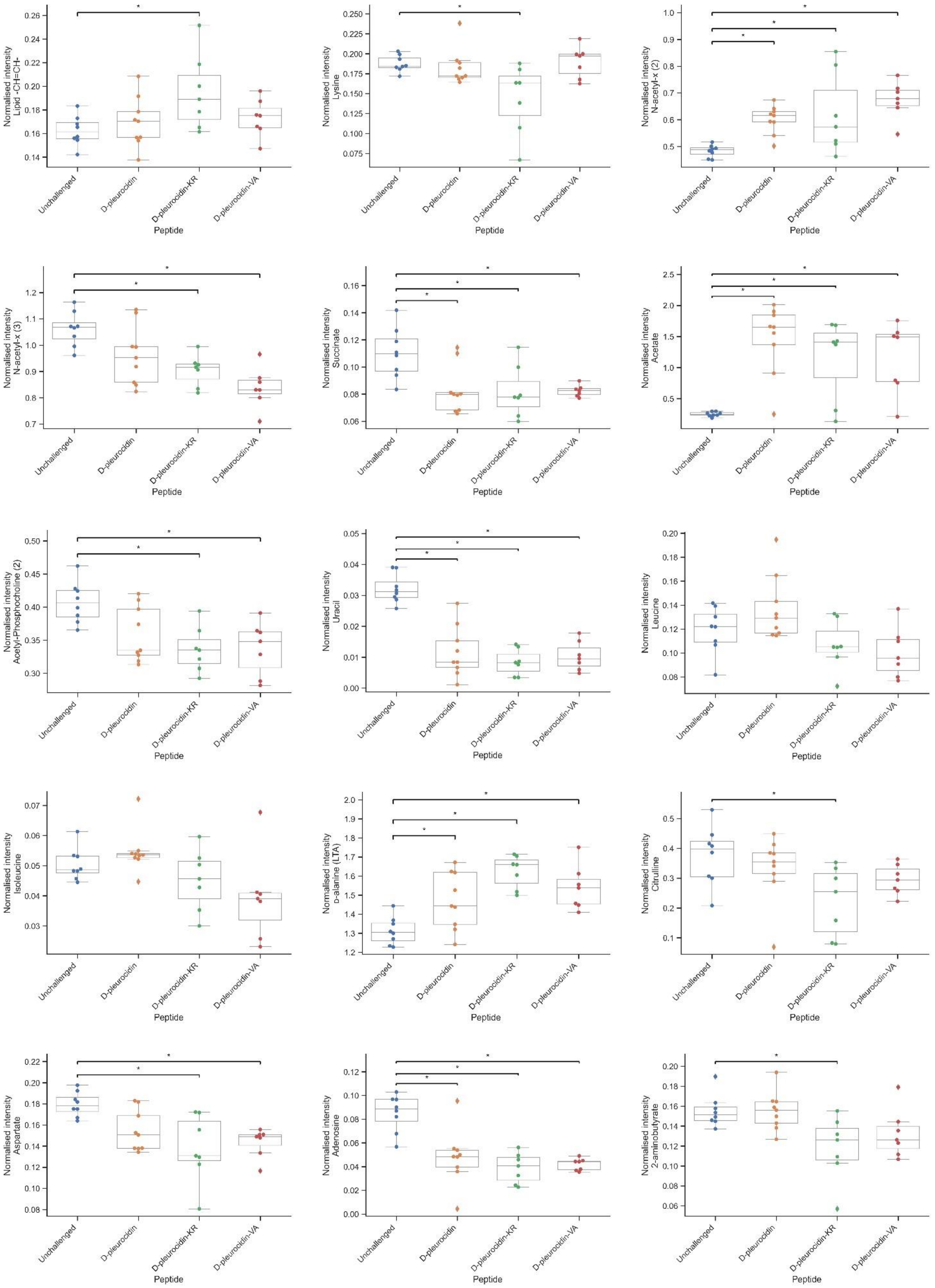


**Supplementary Figure 20 (continued). Modest differences in the response of EMRSA-15 to challenge by pleurocidin analogues in RPMI.** Univariate analysis of individual metabolite variation as observed by $^{1}$H HR-MAS NMR of EMRSA-15 cultured in RPMI and challenged as indicated.

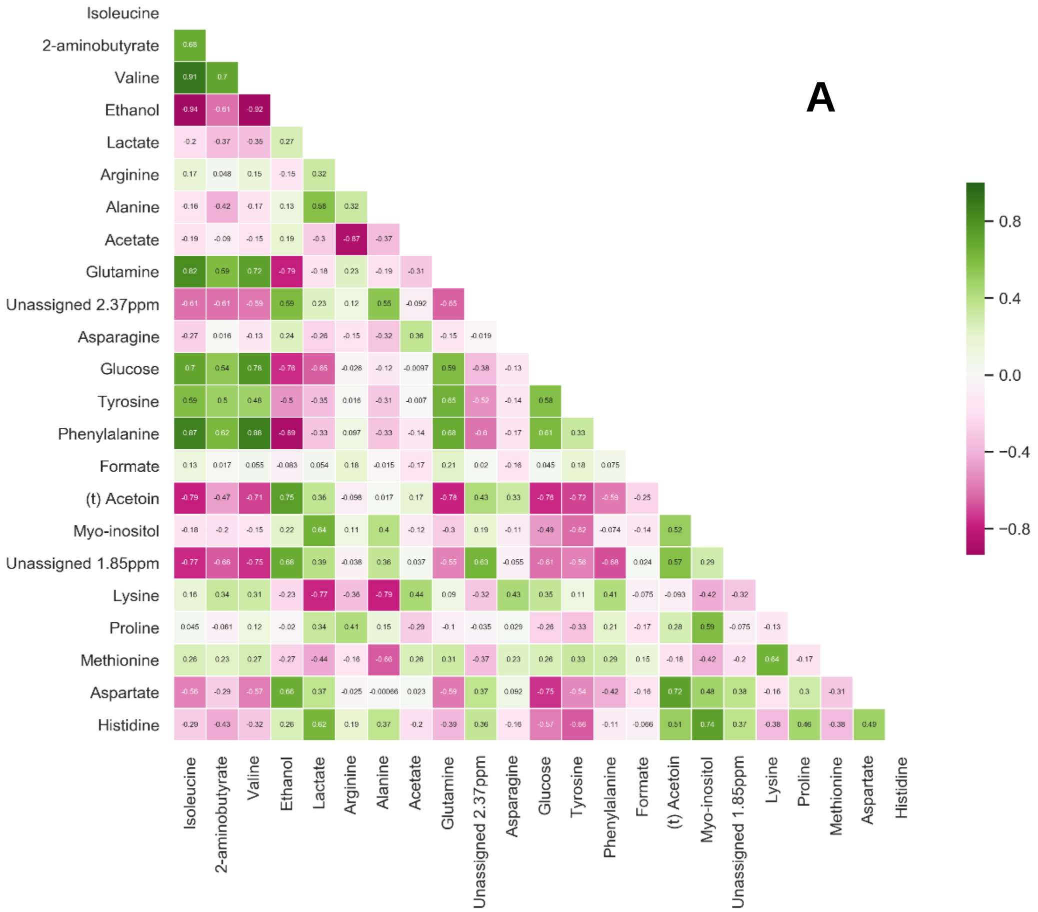


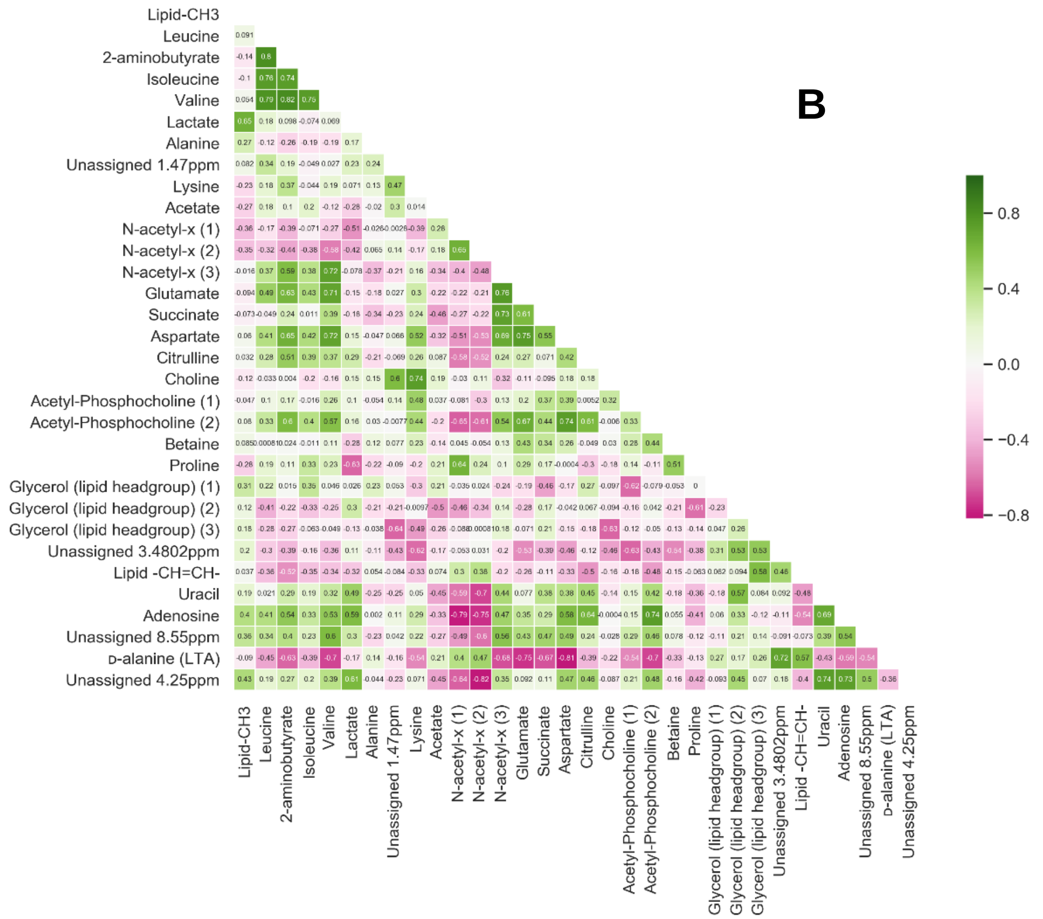


**Supplementary Figure 21. Spearman correlations between metabolites – EMRSA-15 in RPMI.** Spearman correlation matrices are shown for spent media (A) and cellular (B) metabolites across all samples where EMRSA-15 is cultured in RPMI (includes presence/absence of each of three D-pleurocidin analogues).

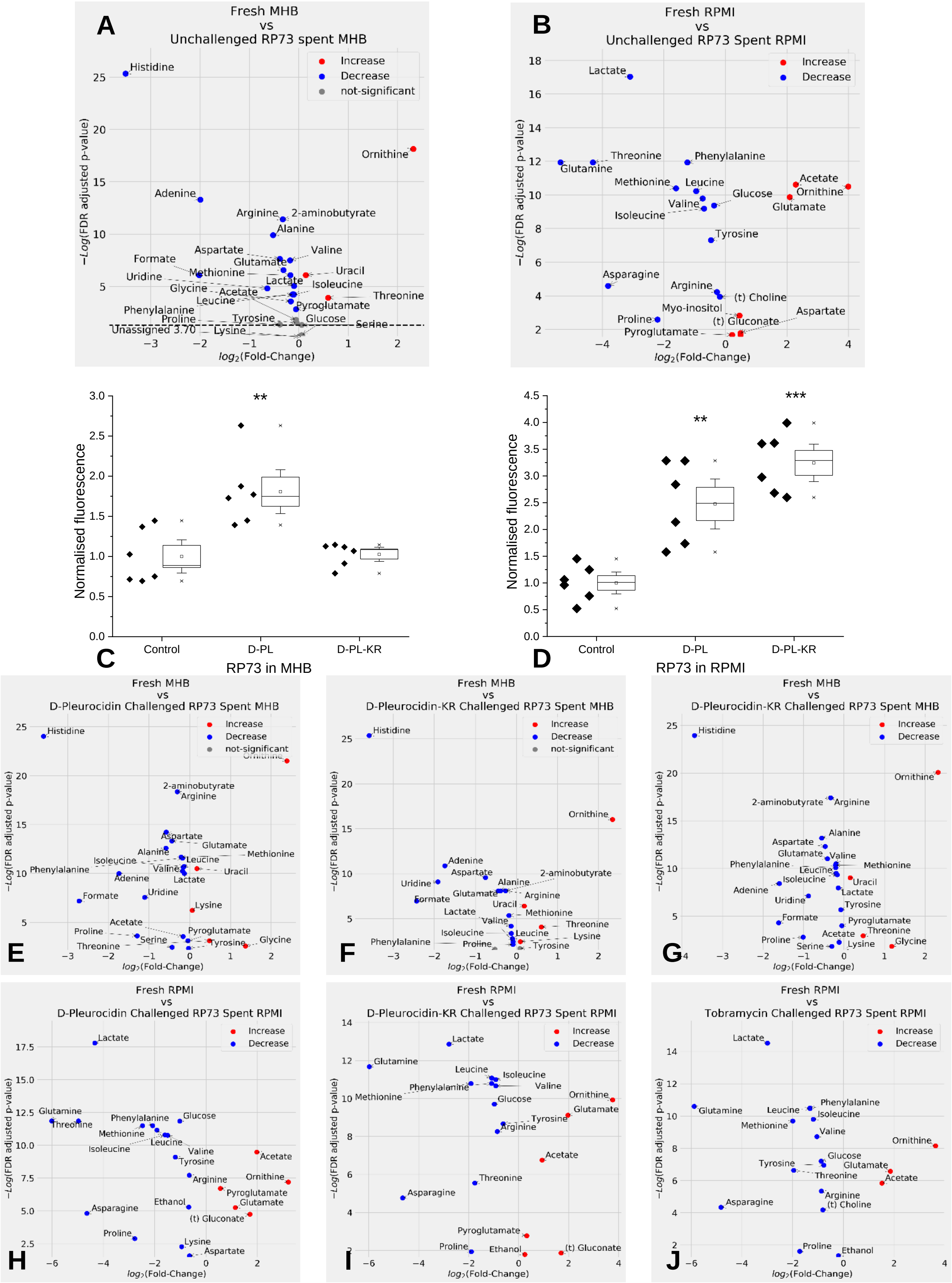


**Supplementary Figure 22. NMR metabolomics identifies altered mechanism of action in D-pleurocidin analogues.** Volcano plots obtained from liquid state $^1$H NMR of spent bacterial culture reveal the metabolic strategy of *P. aeruginosa* RP73 in either MHB (A, E-G) or RPMI (B, H-J) either alone (A, B) or with D-pleurocidin (E, H), D-pleurocidin-KR (F, I) or tobramycin (G, J). Metabolites with non-significant changes are not shown to avoid overlap. Production of ROS/RNS when challenged by antibiotics, as monitored by DCFH-DA, is shown for MHB (C) and RPMI (D). Boxes are 1 SE and bars 1.5 SE.

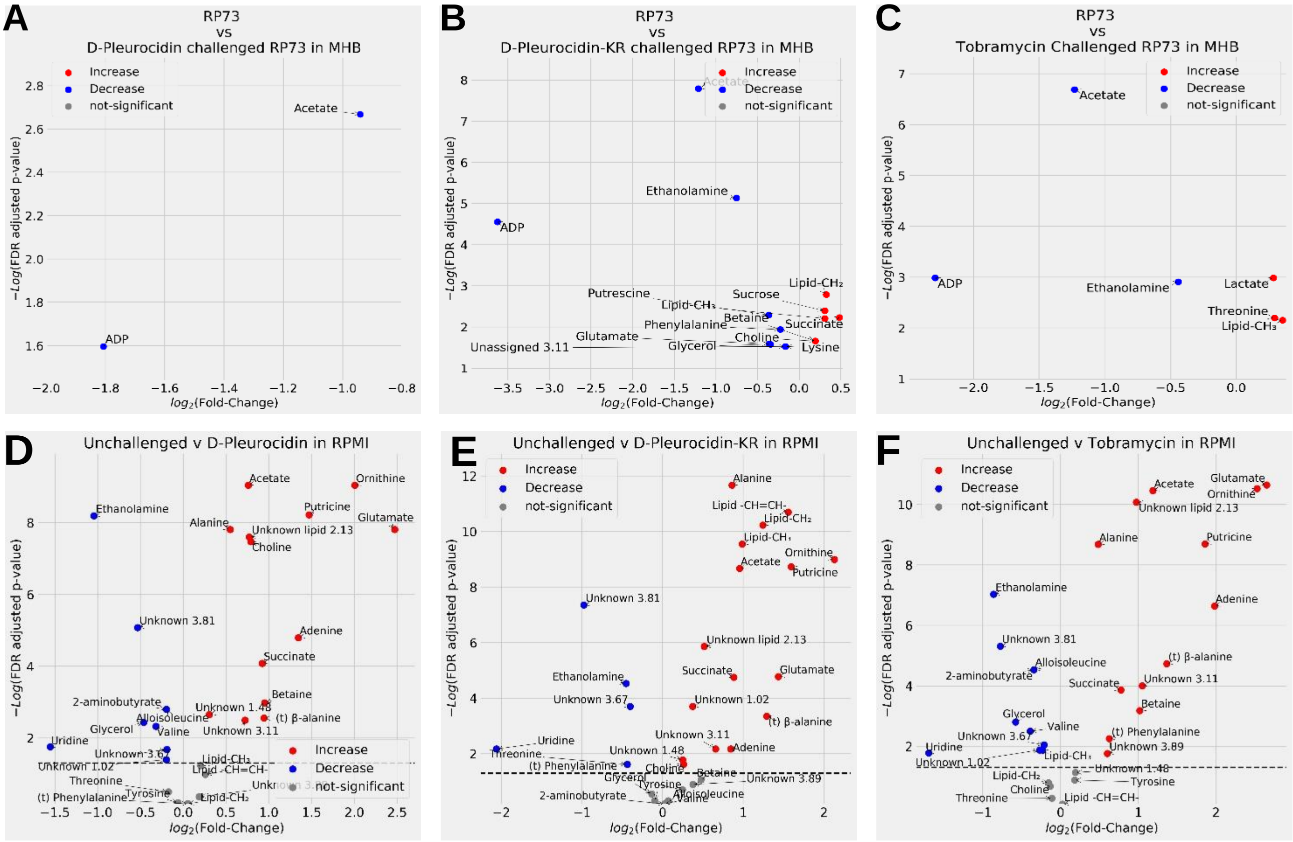


**Supplementary Figure 23. NMR metabolomics identifies altered mechanism of action in D-pleurocidin analogues.** Volcano plots obtained from $^{1}$H HR-MAS NMR of bacterial pellets reveals the effect on cellular metabolites of growth challenge with the indicated antibiotics. Data is shown for *P. aeruginosa* RP73 cultured in MHB (A-C) or RPMI (5% FBS) (D-F). Metabolites with non-significant changes are not shown to avoid overlap in panels A-C.

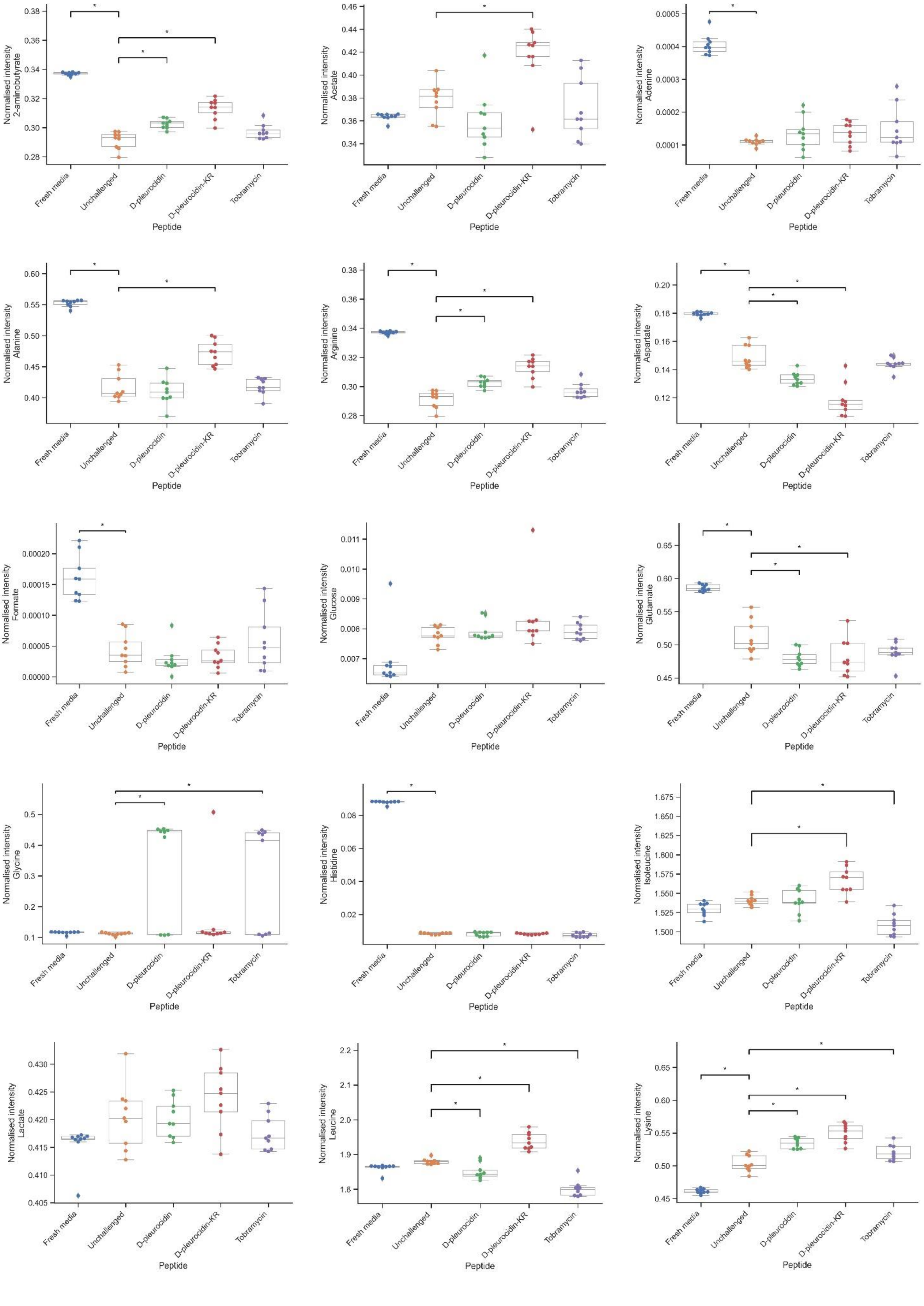


**Supplementary Figure 24. Modest differences in the metabolic strategy of *Pseudomonas aeruginosa* RP73 following challenge by D-pleurocidin analogues in MH broth.** Univariate analysis of individual metabolite variation as observed by $^{1}$H NMR of Mueller-Hinton broth with P. aeruginosa RP73 cultured and challenged as indicated.

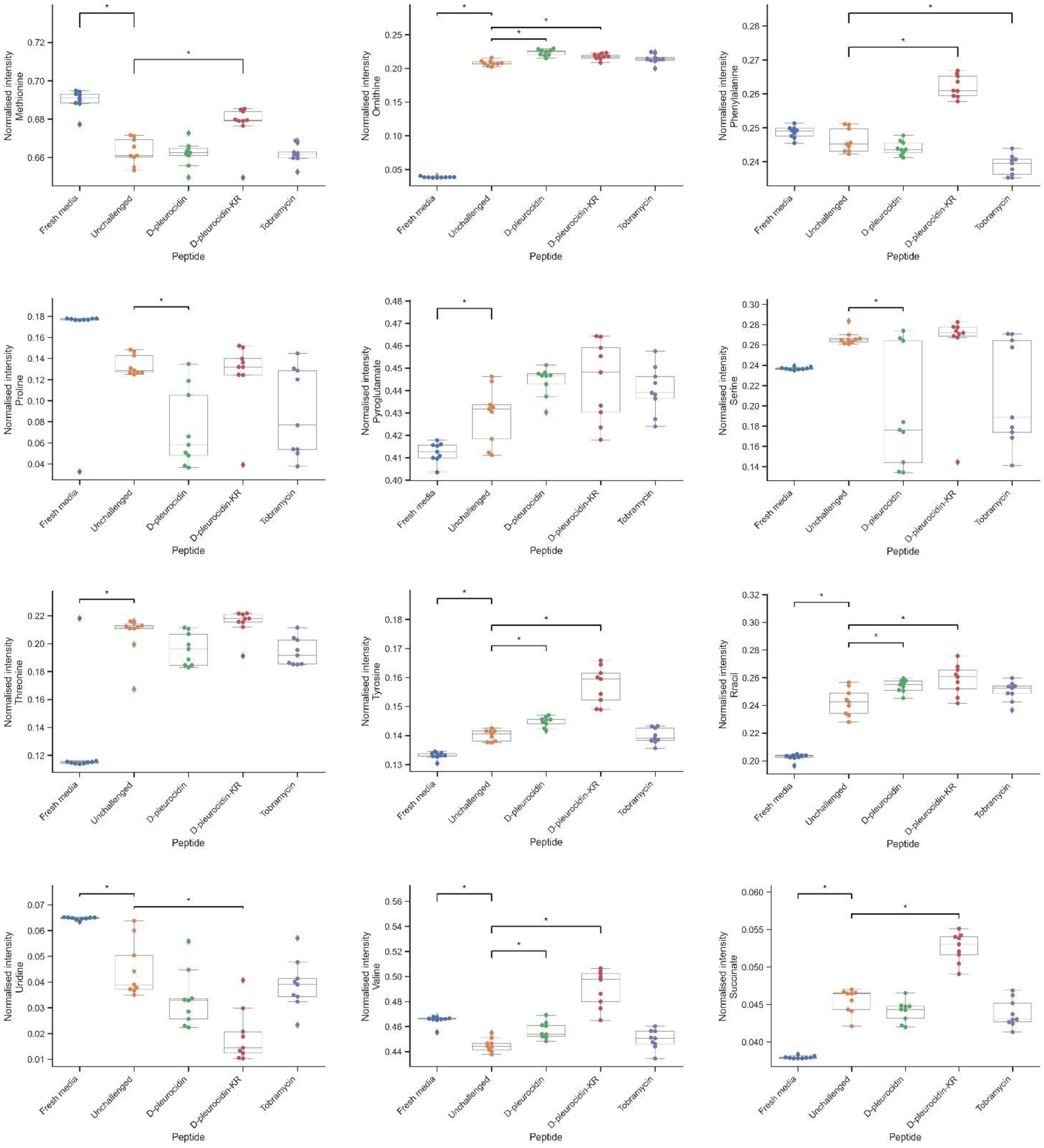


**Supplementary Figure 24 (continued). Modest differences in the metabolic strategy of *Pseudomonas aeruginosa* RP73 following challenge by D-pleurocidin analogues in MH broth.** Univariate analysis of individual metabolite variation as observed by $^{1}H$ NMR of Mueller-Hinton broth with P. aeruginosa RP73 cultured and challenged as indicated.

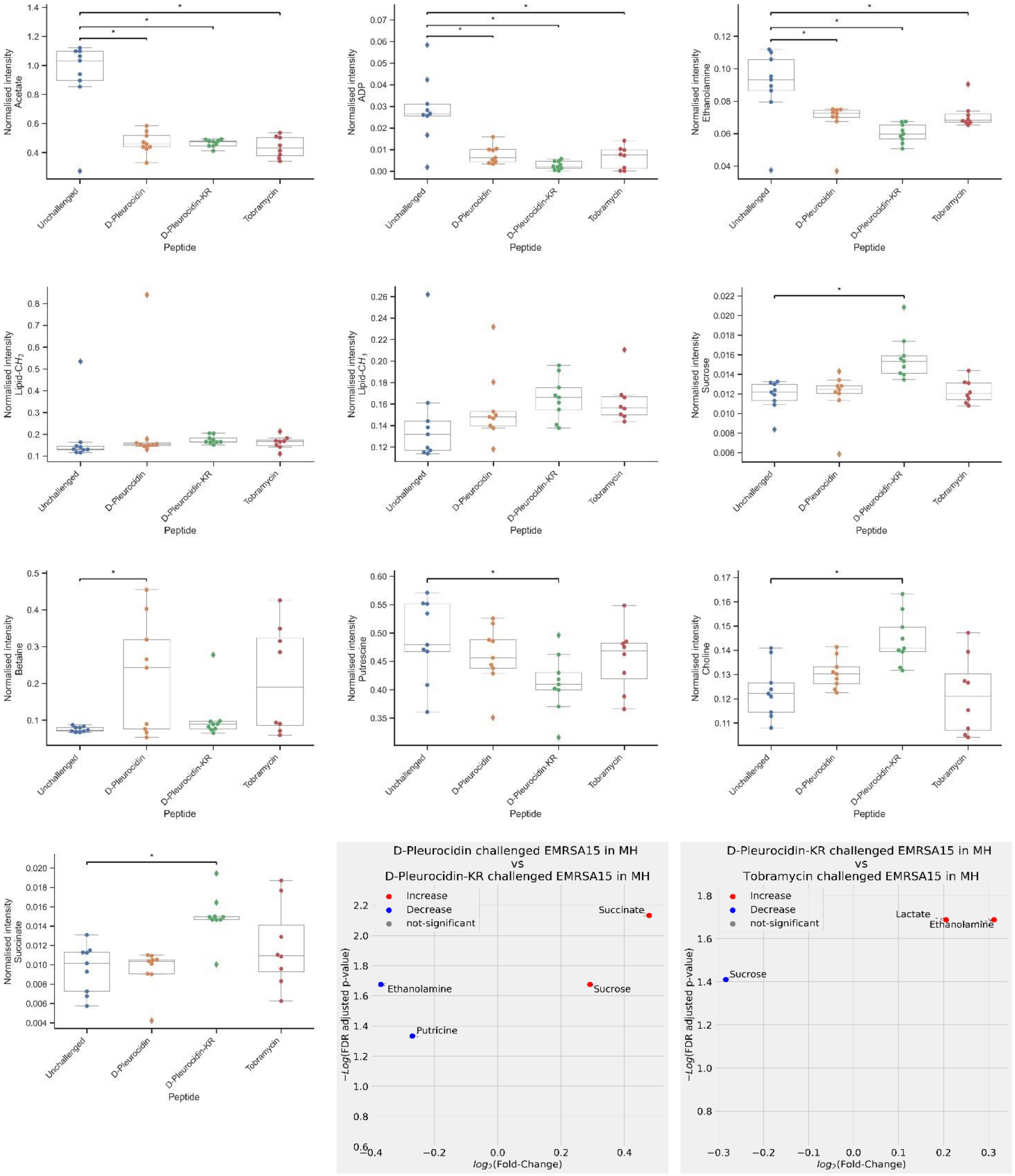


**Supplementary Figure 25. Modest differences in the response of *Pseudomonas aeruginosa* RP73 challenge by pleurocidin analogues and tobramycin in MH broth.** Univariate analysis of individual metabolite variation as observed by $^1$H HR-MAS NMR of *P. aeruginosa* RP73 cultured in Mueller-Hinton broth and challenged as indicated. Other metabolites detected but where there were no significant changes or trends, relative to unchallenged bacteria, include 2-aminobutyrate, alanine, alloisoleucine, glutamate, glycerol, lactate, lysine, ornithine, phenylalanine, threonine, tyrosine, uracil , valine and four unassigned resonances at 1.48, 2.56, 3.11 and 3.26 ppm.

A volcano plot (not shown) comparing D-pleurocidin and tobramycin challenges finds no significant changes in metabolite abundance.

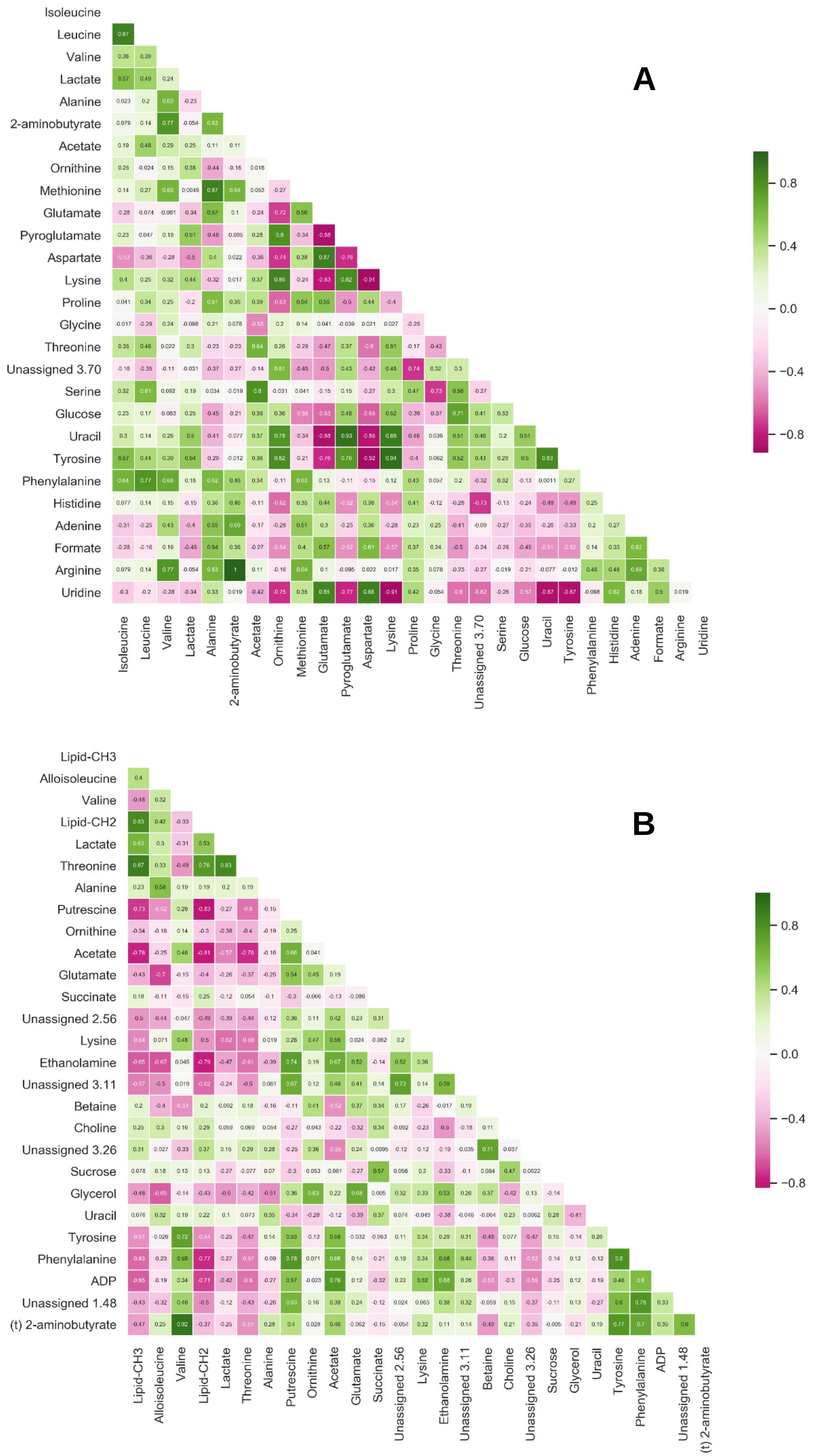


**Supplementary Figure 26. Spearman correlations between metabolites – *P. aeruginosa* RP73 in MHB.** Spearman correlation matrices are shown for spent media (A) and cellular (B) metabolites across all samples where *P. aeruginosa* RP73 is cultured in MHB (includes presence/absence of each of two D-pleurocidin analogues or tobramycin).

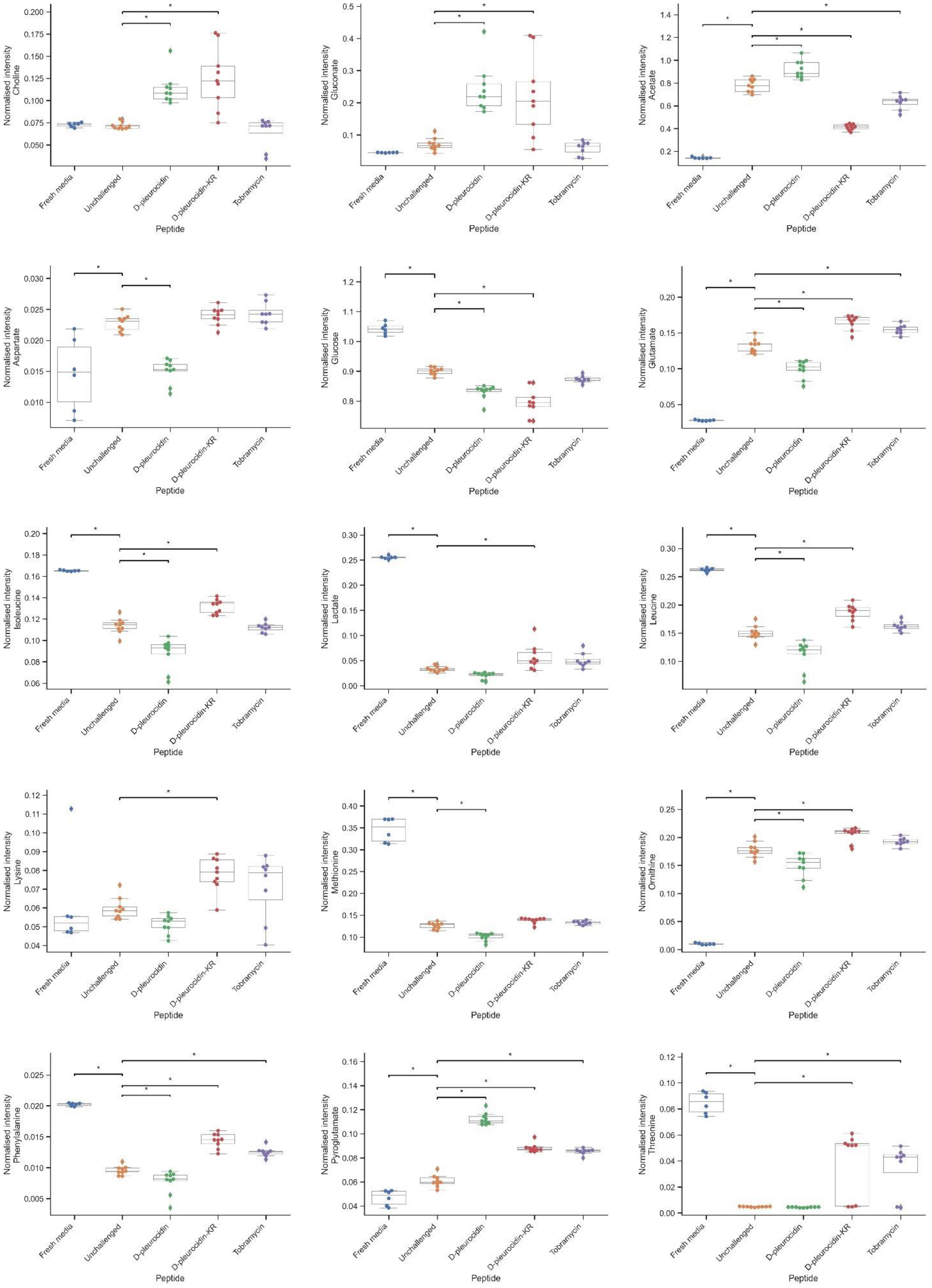


**Supplementary Figure 27. Substantial differences in the metabolic strategy of *P. aeruginosa* RP73 following challenge by D-pleurocidin analogues in RPMI.** Univariate analysis of individual metabolite variation as observed by $^{1}$H NMR of RPMI with *P. aeruginosa* RP73 cultured and challenged as indicated.

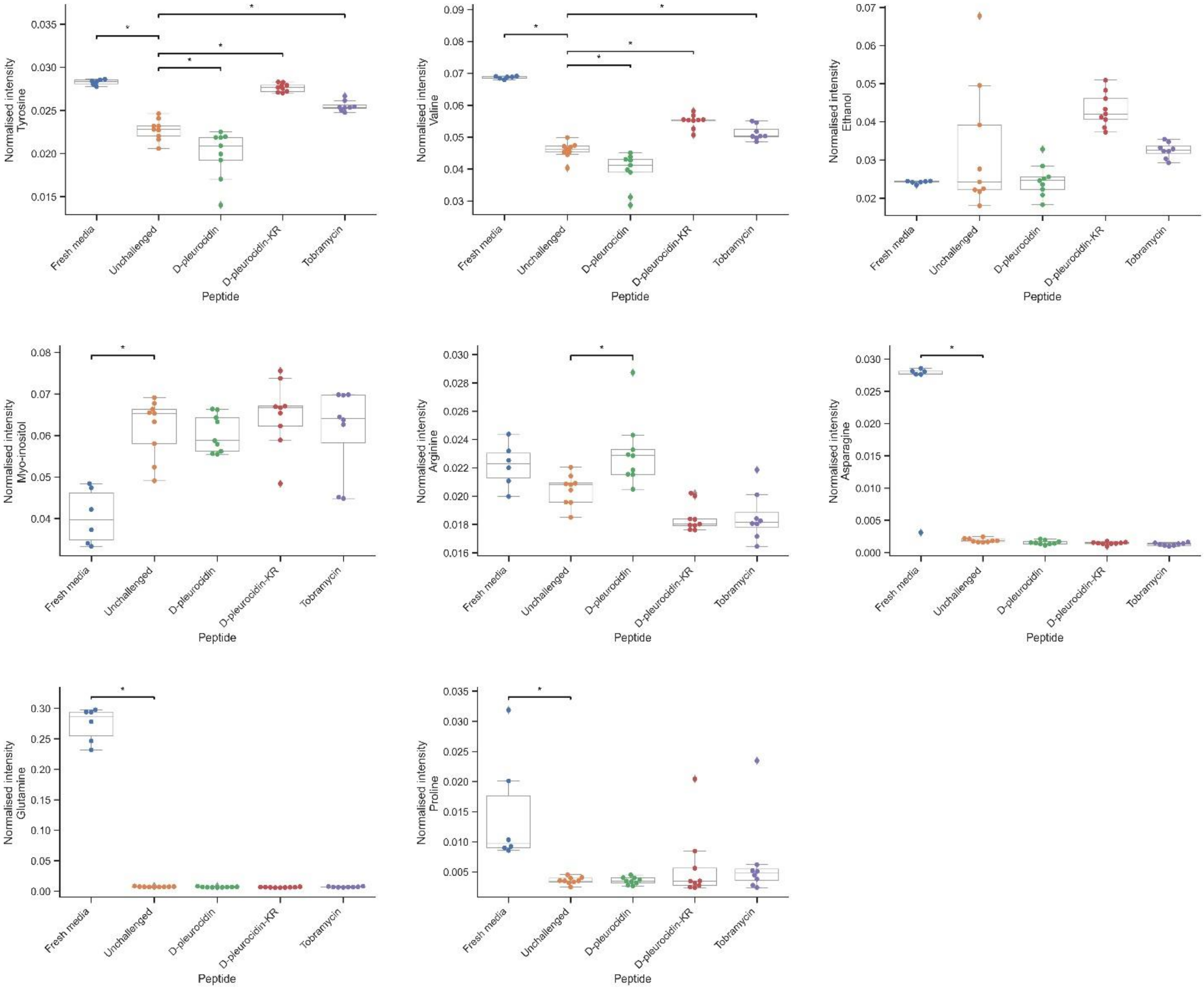


**Supplementary Figure 27 (continued). Substantial differences in the metabolic strategy of *P. aeruginosa* RP73 following challenge by D-pleurocidin analogues in RPMI.** Univariate analysis of individual metabolite variation as observed by $^{1}$H NMR of RPMI with *P. aeruginosa* RP73 cultured and challenged as indicated.

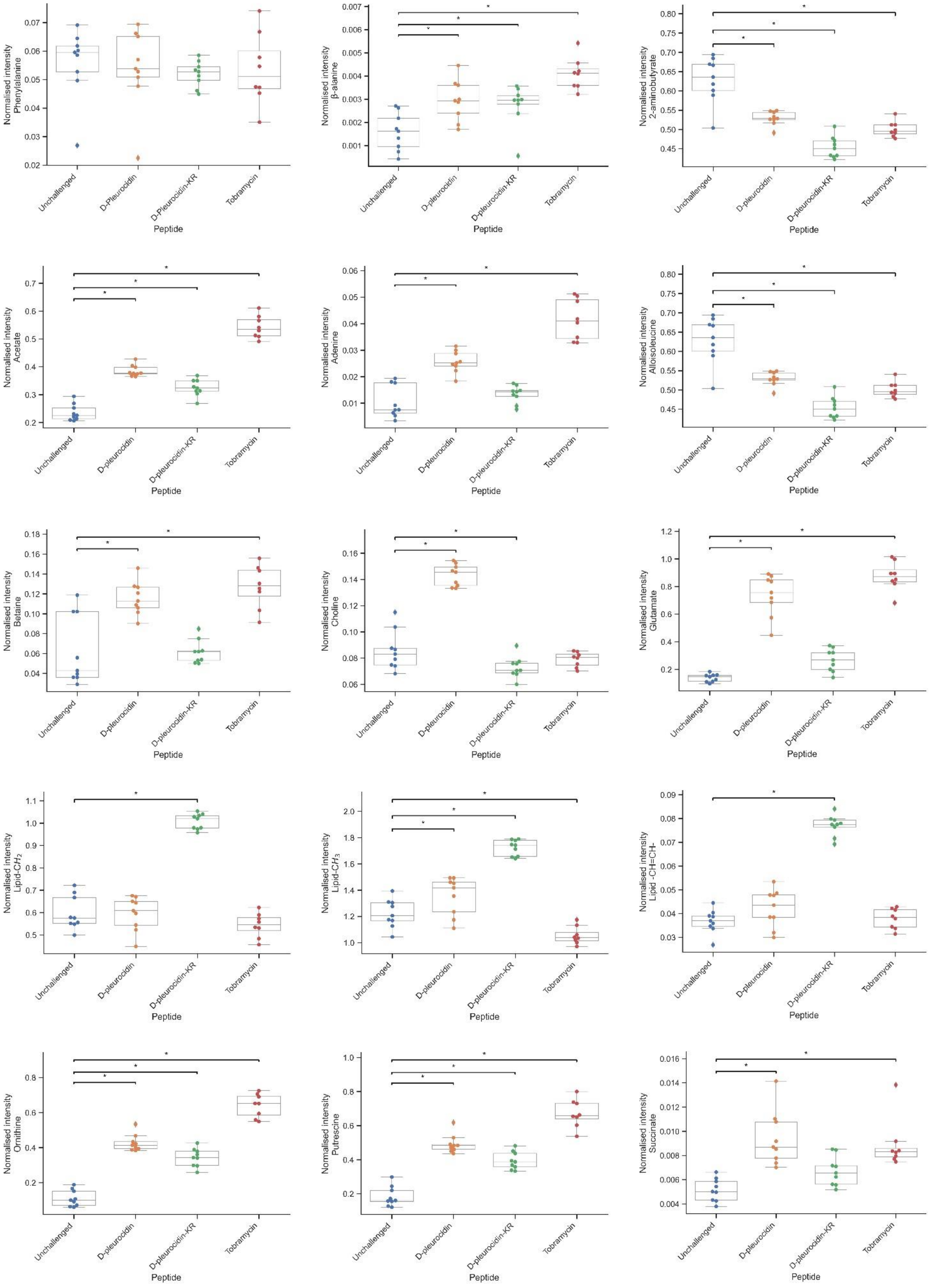


**Supplementary Figure 28. Substantial differences in the response of *Pseudomonas aeruginosa* RP73 challenge by pleurocidin analogues and tobramycin in RPMI.** Univariate analysis of individual metabolite variation as observed by $^{1}$H HR-MAS NMR of *P. aeruginosa* RP73 cultured in RPMI and challenged as indicated. Other metabolites where a change is detected relative to unchallenged bacteria but not between challenges, include ethanolamine, glycerol, L-valine and uridine.

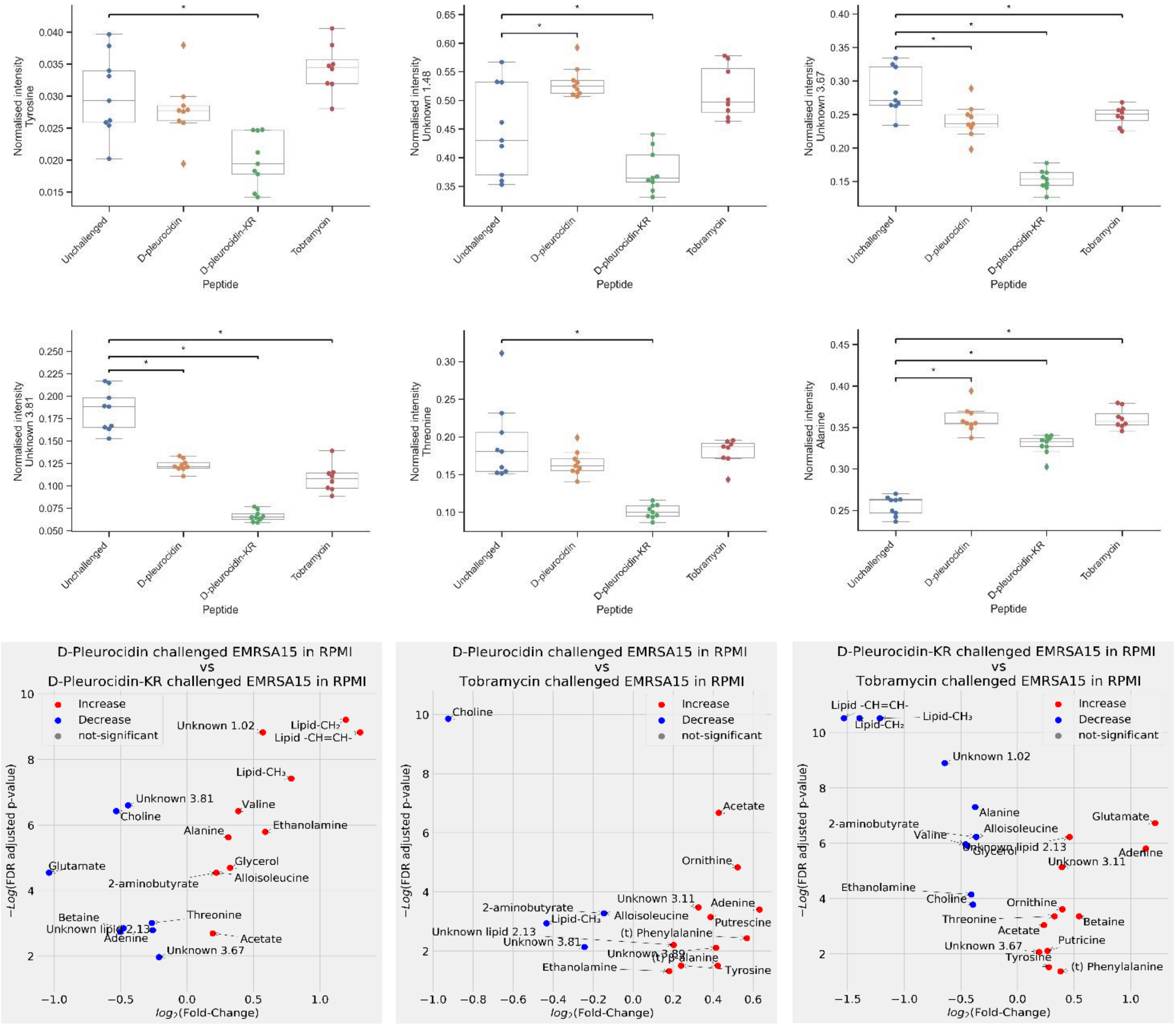


**Supplementary Figure 28 (continued). Substantial differences in the response of *Pseudomonas aeruginosa* RP73 challenge by pleurocidin analogues and tobramycin in RPMI.** Univariate analysis of individual metabolite variation as observed by $^{1}$H HR-MAS NMR of *P. aeruginosa* RP73 cultured in RPMI and challenged as indicated. Other metabolites where a change is detected relative to unchallenged bacteria but not between challenges, include ethanolamine, glycerol, L-valine and uridine. Metabolites with non-significant changes are not shown to avoid overlap.

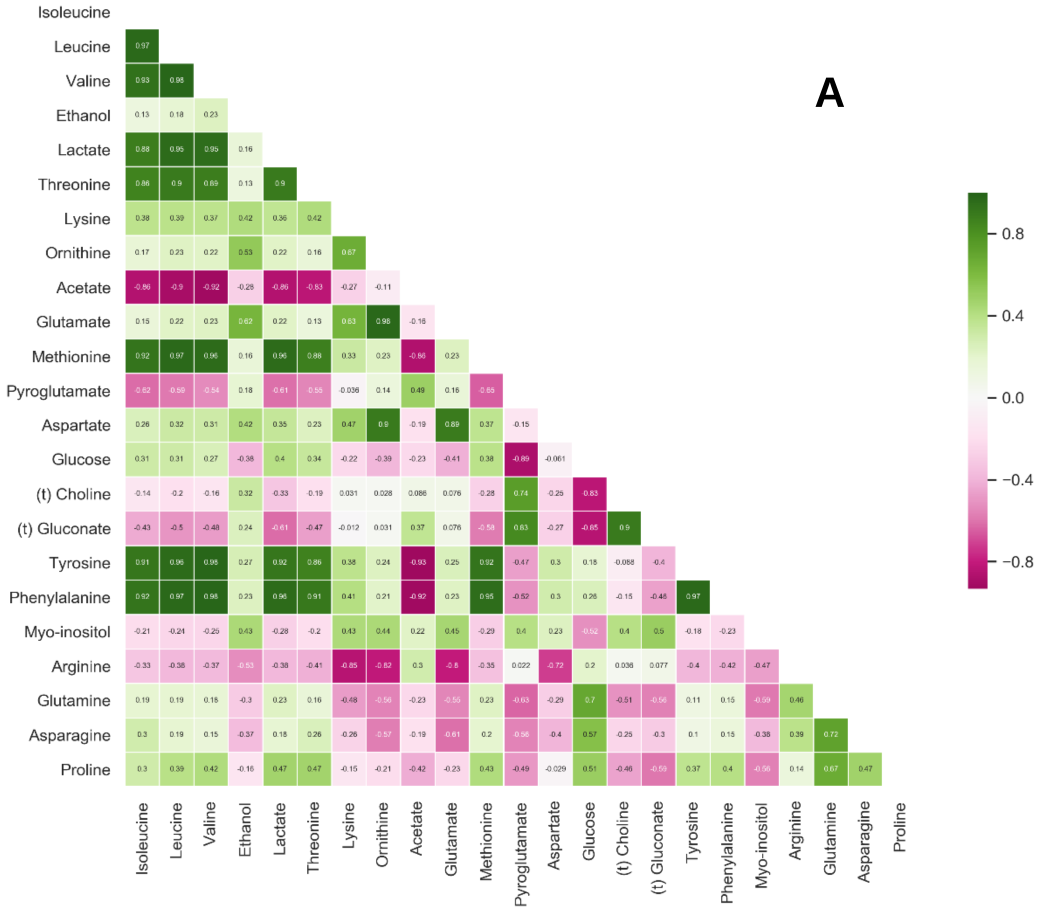


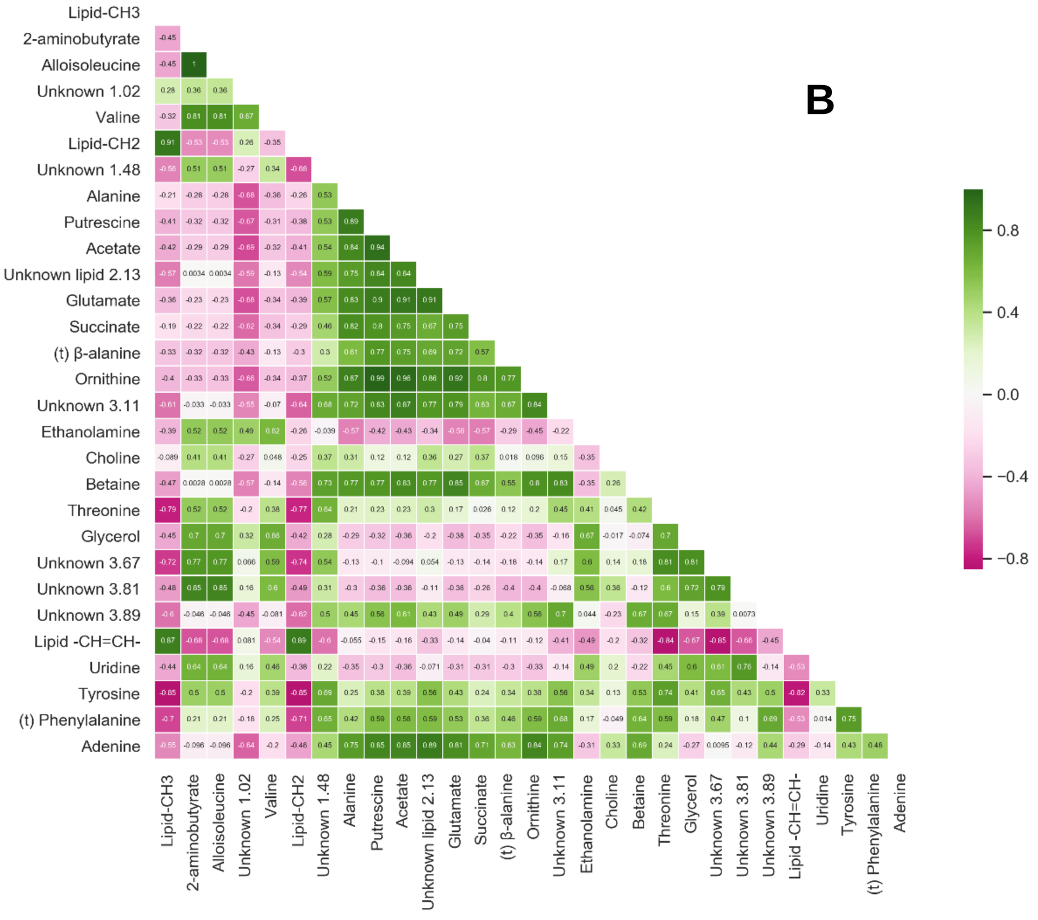


**Supplementary Figure 29. Spearman correlations between metabolites – *P. aeruginosa* RP73 in RPMI.** Spearman correlation matrices are shown for spent media (A) and cellular (B) metabolites across all samples where *P. aeruginosa* RP73 is cultured in RPMI (includes presence/absence of each of two D-pleurocidin analogues or tobramycin).
