# Supplementary material for: A pleurocidin analogue with greater conformational flexibility, enhanced antimicrobial potency and in vivo therapeutic efficacy
Source: Commun Biol. 2020 Nov 27;3:697. doi: 10.1038/s42003-020-01420-3 (PMC7699649; doi:10.1038/s42003-020-01420-3)
Supplement: Supplementary file 3 — Description of Additional Supplementary Files [file 42003_2020_1420_MOESM3_ESM.pdf]

Description of Additional Supplementary Files

File Name: Supplementary Data 1
Description: Source data for main Figures
