# Supplementary material for: A pleurocidin analogue with greater conformational flexibility, enhanced antimicrobial potency and in vivo therapeutic efficacy
Source: Commun Biol. 2020 Nov 27;3:697. doi: 10.1038/s42003-020-01420-3 (PMC7699649; doi:10.1038/s42003-020-01420-3)
Supplement: Supplementary file 5 — Reporting Summary [file 42003_2020_1420_MOESM5_ESM.pdf]

nature research

Corresponding author(s): Dr A. James Mason

Last updated by author(s): Oct 16, 2020

Reporting Summary

Nature Research wishes to improve the reproducibility of the work that we publish. This form provides structure for consistency and transparency in reporting. For further information on Nature Research policies, see Authors & Referees and the Editorial Policy Checklist .

Statistics

For all statistical analyses, confirm that the following items are present in the figure legend, table legend, main text, or Methods section.

n/a	Confirmed	
☐	☒	The exact sample size (*n*) for each experimental group/condition, given as a discrete number and unit of measurement
☐	☒	A statement on whether measurements were taken from distinct samples or whether the same sample was measured repeatedly
☐	☒	The statistical test(s) used AND whether they are one- or two-sided<br>*Only common tests should be described solely by name; describe more complex techniques in the Methods section.*
☐	☒	A description of all covariates tested
☐	☒	A description of any assumptions or corrections, such as tests of normality and adjustment for multiple comparisons
☐	☒	A full description of the statistical parameters including central tendency (e.g. means) or other basic estimates (e.g. regression coefficient) AND variation (e.g. standard deviation) or associated estimates of uncertainty (e.g. confidence intervals)
☒	☐	For null hypothesis testing, the test statistic (e.g. *F*, *t*, *r*) with confidence intervals, effect sizes, degrees of freedom and *P* value noted<br>*Give P values as exact values whenever suitable.*
☒	☐	For Bayesian analysis, information on the choice of priors and Markov chain Monte Carlo settings
☒	☐	For hierarchical and complex designs, identification of the appropriate level for tests and full reporting of outcomes
☒	☐	Estimates of effect sizes (e.g. Cohen's *d*, Pearson's *r*), indicating how they were calculated

Our web collection on statistics for biologists contains articles on many of the points above.

Software and code

Policy information about availability of computer code

Data collection	Gromacs is used for MD simulations and TopSpin (Bruker operating software) is used for NMR data acquisition. PatchControl is the proprietary software from Nanion used for controlling the Port-a-patch. Chirascan CD data and Cary Eclipse fluorescence data were obtained with the respective controlling software supplied with the instrument.
Data analysis	GraphPad Prism v8 was used for data analysis for in vivo data, Origin 8.0 Pro for fluorescence and CD data and Python modules (implementing published mathematical operations) for metabolomics data. Publicly available Gromacs analysis modules were used for MD data analysis and in-house plotting scripts which can be made available on request.

For manuscripts utilizing custom algorithms or software that are central to the research but not yet described in published literature, software must be made available to editors/reviewers. We strongly encourage code deposition in a community repository (e.g. GitHub). See the Nature Research guidelines for submitting code & software for further information.

Data

Policy information about availability of data

All manuscripts must include a data availability statement. This statement should provide the following information, where applicable:

- Accession codes, unique identifiers, or web links for publicly available datasets
- A list of figures that have associated raw data
- A description of any restrictions on data availability

Structural coordinates were deposited in the Protein Data Bank (www.rcsb.org) and Biological Magnetic Resonance Bank (BMRB; www.bmrb.wisc.edu) under accession codes of 6RSF and 6RSG (PDB) and 34404 and 34405 (BRMB) for pleurocidin-KR and pleurocidin-VA respectively. In addition to the structural coordinates the datasets generated during and/or analysed during the current study are available from the corresponding author on reasonable request.

Field-specific reporting

Please select the one below that is the best fit for your research. If you are not sure, read the appropriate sections before making your selection.

- [x] Life sciences
- [ ] Behavioural & social sciences
- [ ] Ecological, evolutionary & environmental sciences

For a reference copy of the document with all sections, see nature.com/documents/nr-reporting-summary-flat.pdf

Life sciences study design

All studies must disclose on these points even when the disclosure is negative.

Sample size	Samples sizes were chosen as an n = 6 per control group and n = 4 for treatment group in each individual experiment which was consequently, independently repeated once to give aggregated n = 12 for control groups and n = 8 for treatment groups, as previous research as demonstrated this to be sufficient to detect accurate changes. This is was performed to sit in line with the 3Rs for animal research
Data exclusions	Data was only excluded from animals where clear issues had been detected, ie: issues identified with infection inoculation or treatment injections
Replication	In vivo studies were repeated over two individual experiments which demonstrated high levels of reproducibility
Randomization	Animals were allocated to groups by the cages, due to logistical constraints in the animal research unit
Blinding	Data groups were blinded during data collection

Reporting for specific materials, systems and methods

We require information from authors about some types of materials, experimental systems and methods used in many studies. Here, indicate whether each material, system or method listed is relevant to your study. If you are not sure if a list item applies to your research, read the appropriate section before selecting a response.

Materials & experimental systems

n/a	Involved in the study	
x		Antibodies
x		Eukaryotic cell lines
x		Palaeontology
	x	Animals and other organisms
x		Human research participants
x		Clinical data

Methods

n/a	Involved in the study	
x		ChIP-seq
x		Flow cytometry
x		MRI-based neuroimaging

Animals and other organisms

Policy information about studies involving animals; ARRIVE guidelines recommended for reporting animal research

Laboratory animals	Male C57/Bl6 mice were used for this study at an age of 8-10 weeks
Wild animals	The study did not involve wild animals.
Field-collected samples	The study did not involve samples collected from the field.
Ethics oversight	All animal studies were performed under Project License P11C06933 approved under the UK Animal (Scientific Procedures) Act 1986 2012 Revision

Note that full information on the approval of the study protocol must also be provided in the manuscript.
